# Antimicrobial Activity and Degradation Ability Study on Nanoparticle-Enriched Formulations Specially Designed for the Neutralization of Real and Simulated Biological and Chemical Warfare Agents

**DOI:** 10.3390/ph15010097

**Published:** 2022-01-14

**Authors:** Raluca-Elena Ginghina, Gabriela Toader, Munizer Purica, Adriana-Elena Bratu, Claudiu Lazaroaie, Tudor-Viorel Tiganescu, Ramona-Elena Oncioiu, George-Ovidiu Iorga, Florina-Lucica Zorila, Mihai Constantin, Gabriel Craciun, Florin Comanescu, Cosmin Romanitan

**Affiliations:** 1 Research and Innovation Center for CBRN Defense and Ecology, 225 Oltenitei Ave., 041327 Bucharest, Romania; ginghinaraluca@gmail.com (R.-E.G.); adriana.bratu@nbce.ro (A.-E.B.); claudiu@nbce.ro (C.L.); ramona.oncioiu@nbce.ro (R.-E.O.); ovidiu.iorga@nbce.ro (G.-O.I.); 2 Military Technical Academy “Ferdinand I”, 39-49 George Cosbuc Boulevard, 050141 Bucharest, Romania; tiganescu.viorel.t@gmail.com; 3 National Institute for R&D in Microtechnologies, 126A Erou Iancu Nicolae Street, 077190 Voluntari, Romania; gabriel.craciun@imt.ro (G.C.); florin.comanescu@imt.ro (F.C.); cosmin.romanitan@imt.ro (C.R.); 4 Horia Hulubei National Institute for Physics and Nuclear Engineering, 30 Reactorului Street, 077125 Magurele, Romania; florina.zorila@nipne.ro (F.-L.Z.); mconstantin@nipne.ro (M.C.); 5 Department of Genetics, Faculty of Biology, University of Bucharest, 91-95 Splaiul Indepententei, 050095 Bucharest, Romania

**Keywords:** *Bacillus anthracis*, antimicrobial activity, mustard gas, soman, decontamination, nanoparticles, ZnO, TiO_2_, zeolite

## Abstract

The present work reveals a comprehensive decontamination study on real and simulated biological and chemical warfare agents (BCWA). The emphasis was on evaluating the antimicrobial activity against real biological warfare agents, such as *Bacillus anthracis*, and also the capacity of neutralizing real chemical warfare agents, such as mustard gas or soman, by employing three different types of organic solutions enriched with ZnO, TiO_2_, and zeolite nanoparticles, specially designed for decontamination applications. The capacity of decontaminating BCWA was evaluated through specific investigation tools, including surface monitoring with the swabs method, minimum inhibitory (MIC) and minimum bactericidal concentration (MBC) evaluations, time-kill tests for microorganisms, and GC-MS for monitoring chemical agents on different types of surfaces (glass, painted metal, rubber, and cotton butyl rubber). These tests revealed high decontamination factors for BCWA even after only 10 min, accomplishing the requirements imposed by NATO standards. At the completion of the decontamination process, the formulations reached 100% efficacy for *Bacillus anthracis* after 10–15 min, for soman after 20–30 min, and for mustard gas in an interval comprised between 5 and 24 h depending on the type of surface analyzed.

## 1. Introduction

Biological and chemical warfare agents (BCWA) for mass destruction have been used in military conflicts and, unfortunately, the risk of being used by terrorist organizations is imminent [1]. Both biological and chemical warfare agents can be designated as weapons of terror against civilians or weapons of intimidation for the soldiers [2].

The use of biological warfare agents became more refined during the 19th century due to the advancements of modern microbiology which made possible the isolation and production of considerable stocks of specific pathogens [3,4]. During the first World War, the use of *Bacillus anthracis* (anthrax) and *Pseudomonas pseudomallei* (glanders) as biological weapons was reported [2,4]. In the same conflict (World War I), chemical weapons were also employed, mustard gas accounting for 80% of all the chemical casualties [2].

Many pathogens cause health diseases; however, only few of them possess characteristics that allow them to be used as bioweapons. A biological agent used as a bioweapon has several features, including high lethality, communicability, fast and predictable action, ability to survive in the environment if it encounters its host, and resistance to destruction with air, water, and food purification methods [5]. Biological agents are usually designed to be susceptible to treatments or vaccines, which are only available to those who perform the attack and not accessible to the victims [5]. Modern bioengineering can hypothetically allow for accomplishing these malicious intentions by creating, for example, antibiotic-resistant strains of anthrax, reducing the time of incubation of smallpox, or combining agents such as Ebola virus and anthrax in order to develop new diseases [5].

Analogous weapons consisting of chemical warfare agents (CWA) that are usually capable of rapid incapacitation, sudden death, or permanent harmful effects on health [6] were intended for use in warfare as “mass destruction weapons”. CWAs can be classified into nerve, blistering, choking, incapacitating/behavior altering, and blood/asphyxiant agents [2,6,7,8,9,10]. The main existing chemical threats involve easily synthesized chemical agents theoretically manufacturable on a large scale, such as nitrogen and sulfur mustards (e.g., yperite) or organophosphorus nerve agents (e.g., soman, sarin, tabun, or Vx) [7]. From these various types of CWAs, nerve agents are considered one of the most lethal chemical weapons due to their phosphorylating mode of action derived from their organophosphorus structure, which leads to mammalian acute toxicity and/or death [10].

Unfortunately, the ease of production and dissemination of BCWAs makes possible the idea of being used by terrorist attacks against civilians.

After their discovery, the inhuman aspect of these BC weapons was soon recognized and in 1925, the Geneva Protocol for the interdiction of the use of chemical or bacteriological methods of warfare was signed [2,9]. This protocol was followed by the implementation of “The Convention on the Prohibition of the Development, Production, Stockpiling, and Use of Chemical Weapons And on Their Destruction (CWC)” [11] and “The Convention on the Prohibition of the Development, Production and Stockpiling of Bacteriological (Biological) and Toxin Weapons and on their Destruction (BWC)” [12].

In this context, biological and chemical defense research implies finding solutions for the early identification of BCWA threats and also developing efficient countermeasures in the case of BCWA attacks. The conventions, namely CWC and BWC, did not forbid research in this field because numerous cases of BCWA utilization were reported over time [2,7,8,13,14,15], thus it is still indispensable to develop versatile methods for “on-site” efficient and sustainable neutralization of biological and chemical warfare agents. Due to the severe toxicity of the real biological and chemical warfare agents’ class, various simulants [1] are sometimes employed in research studies. Even if the use of non-pathogenic microorganisms as simulants of Biological Warfare Agents (BWA-S) could be useful in the initial phase of the development of a new decontamination technology, BWA-S imply, however, some limitations because they may have in common some of the properties of the biological warfare agents, but they also have different antigens, proteomes, and genomes [5]. The simulants of chemical warfare agents (CWA-S) present the same advantages and disadvantages as BWA-S, ensuring proper safe circumstances for preliminary screenings of the decontamination efficacy. Nevertheless, it is still essential to perform extensive tests on real warfare agents, too, for the proper design of the new decontamination formulations and for an objective evaluation of their performances.

When encountering a BCWA event, it is crucial to immediately decontaminate the exposed area to an acceptable level, thus victims can be located and treated [1,3]. Further decontamination steps may be required to reestablish the functionality of facilities or equipment. For large contaminated zones, rapid and effective methods for the neutralization and removal of toxic biological and chemical warfare agents is imperative to restore the combat effectiveness of the strategic elements (equipment, facilities, and personnel) [1,16].

During the past decades, various decontamination methods were developed by researchers for the neutralization of BCWA. Hydrolysis or oxidation by aqueous-based decontamination solutions proved their efficiency against BCWA but the quantity of wastewater generated post-decontamination is significant and requires expensive disposal. Limiting effluent volumes represents an imperative goal [7]. Consequently, organic neutralizing solutions (e.g., alcoholic) offer attractive approaches because this method involves lower amounts of post-decontamination waste [7].

Modern decontamination techniques also involve the use of the benefits brought by nanotechnology. In this regard, choosing the proper active nanosized ingredients could lead to higher BCWA decontamination performances. Several studies revealed the positive influence of various types of nanostructures on the neutralization of BCWA [10,17,18,19,20,21,22]. Numerous studies revealed that the presence of metal or metal oxides into a decontamination system favors achieving a selective and low temperature degradative process of BCWA [10].

From the wide variety of nanoparticles described in literature as active agents for BCWA neutralization, the antibacterial activity [20,23,24] of zinc oxide nanoparticles and their capacity for neutralizing chemical warfare agents [25,26,27] received significant interest. ZnO is considered a bio-safe substance that possesses photo-oxidizing and photocatalysis effects on chemical and biological species [24]. Bactericidal and bacteriostatic mechanisms of ZnO nanoparticles can be attributed to the presumable generation of reactive oxygen species (ROS) and to the permeation of the cell membrane to toxic dissolved zinc ions [24]. Zinc oxide nanomaterials have been also reported to be successfully used as reactive sorbents for the detoxification of sulfur mustard [27] or nerve agents [26].

Another nanomaterial which attracted great interest for BCWA inactivation is represented by titanium dioxide [28,29,30,31]. TiO_2_ is a remarkable photocatalyst widely used for its antibacterial action due to its high photosensitivity, high efficiency, non-toxic nature, strong oxidizing ability, relative cheapness, and chemical stability [31]. Titanium dioxide nanoparticles are in fact one of the most studied materials for antimicrobial applications due to its bactericidal photocatalytic activity, safety, and self-cleaning properties. The mechanism of antimicrobial action of TiO_2_ is commonly associated to reactive oxygen species (ROS) with high oxidative abilities which affect bacterial cells by different mechanisms, leading to their death [32]. The increased surface area of TiO_2_ nanoparticles makes them suitable as antimicrobial agents because it increases the possibility of interaction with pathogenic bacteria. Moreover, their nanometric size allows them to easily enter through the membrane of the targeted microorganisms and damage their structure from the interior [30]. The photocatalytic activity of TiO_2_ brings multiple advantages also for the neutralization of chemical warfare agents through photodegradation mechanisms and the generation of ROS [18]. The large specific surface of nanosized titanium dioxide and the presence of surface hydroxyl groups allows for the efficient decontamination of chemical agents such as sulfur mustard and sarin, forming non-toxic products such as thiodiglycol and isopropyl methyl phosphonic acid, respectively [18].

A unique class of materials, which is widely utilized as adsorbents for various types of contaminants, is represented by zeolites. Their well-defined porous structure recommends them for various applications in catalysis, ion exchange, and adsorption processes [33]. Clinoptilolite (Cp) is a type of zeolite suitable for BCWA decontamination applications due to its distinctive features and advantages such as its low cost, availability, and most abundant natural zeolite [34]. Several toxicological studies proved that zeolites are non-toxic [33]. Even so, zeolites can be ion exchanged with Ag(^+^), Zn(^2+^), or Cu(^2+^) ions, or they can be employed in a decontamination solution containing other active ingredients to acquire antimicrobial properties or ion-releasing characteristics for providing prolonged or stronger activity [35]. Ag^+^ ion-exchanged clinoptilolite zeolite has been reported to promote CWA-S (e.g., chloroethyl ethyl sulfide and dimethyl methyl phosphonate) adsorption and degradation [34].

Considering all the drawbacks of the existing decontamination methods and inspired by the recent findings in the field of BCWA, this paper proposes novel solutions, consisting of innovative nanoparticle-enriched formulations specially designed for the inactivation of real and simulated biological and chemical warfare agents. The novelty of this work consists of the multivalent character of the proposed decontamination formulations (suitable for both biological and chemical warfare agents) and the comprehensive evaluation of their efficacy against one real biological agent (*Bacillus anthracis*) as well as two real warfare agents (sulfur mustard and soman). Since the studies found in literature were carried out mostly on simulants, as far as we are concerned, we can affirm that this paper is the first one comprising extensive decontamination studies on real biological and chemical warfare agents performed on various types of surfaces according to NATO standards. The new formulations herein reported are comprised of an active organic solution as dispersion media and one of three types of nanosized adsorbents, namely ZnO, TiO_2_, and zeolite, employed for the enhancement of the decontamination performances of the organic solution (higher antimicrobial activity and higher capacity for neutralizing chemical warfare agents).

## 2. Results

**Figure 1:**
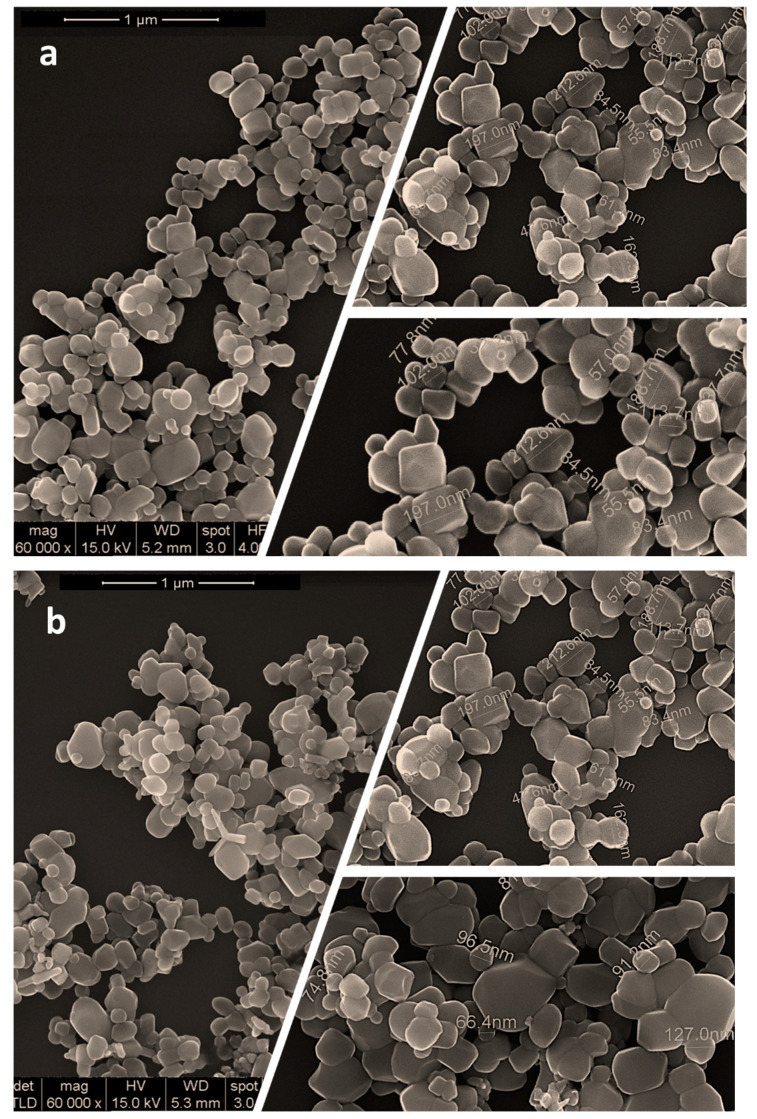

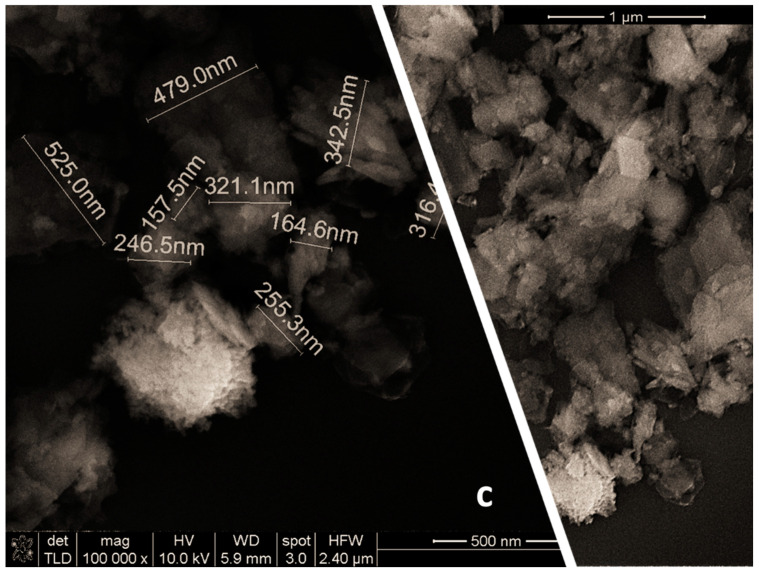
SEM images of the synthesized nanoparticles (**a**) ZnO, (**b**) TiO_2_, and (**c**) zeolite.

The decontamination solutions employed in this study were comprised of three types of nanoparticles: ZnO, TiO_2_, and zeolite (the sample IDs are summarized in Table 1). As it will be further discussed in detail, the presence of these nanosized adsorbents in the organic solution proved that they could enhance the BCWA decontamination capacity. Figure 1 illustrates SEM images captured on each type of the three synthesized nanoparticles.

**Figure 2:**
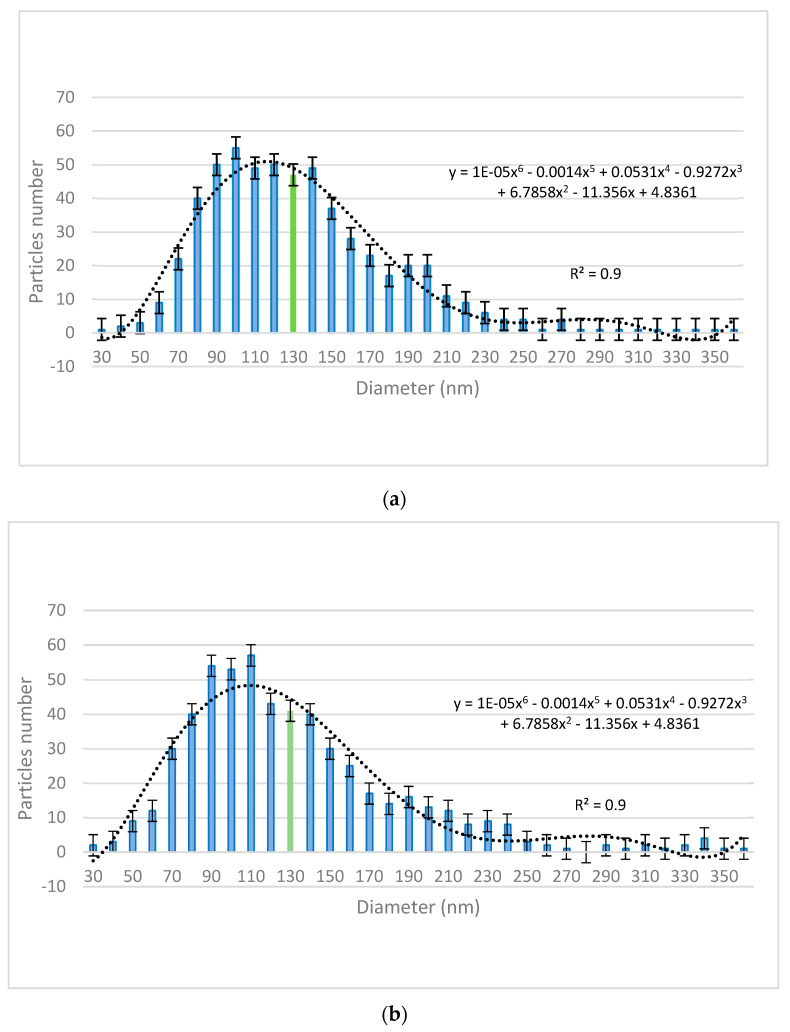

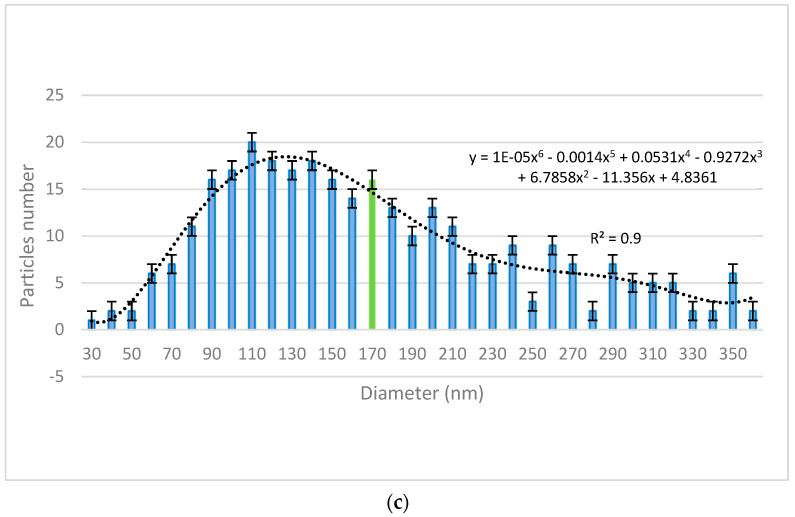
Size distribution of the synthesized nanoparticles: (**a**) ZnO (mean size value obtained: 134.2 nm), (**b**) TiO_2_ (mean size value obtained: 131.2 nm), and (**c**) zeolite (mean size value obtained: 171.1 nm).

The size distribution of the nanoparticles was assessed using VegaTC software provided by Tescan and are displayed in Figure 2. The mean particle diameter is highlighted in the histograms (green bars).

**Figure 3:**
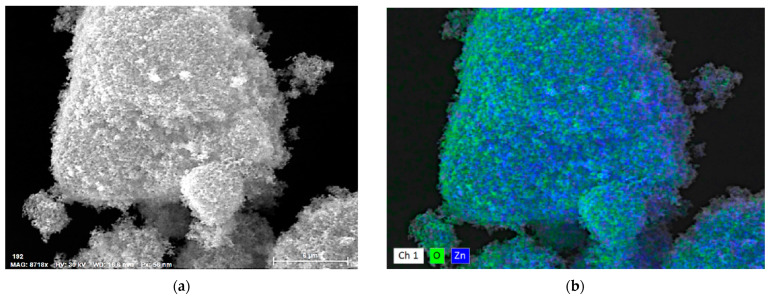

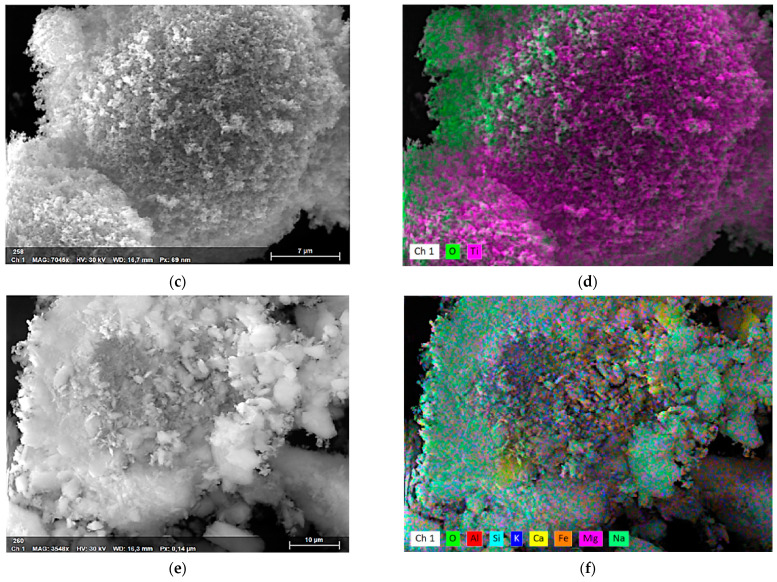
SEM and EDX mapping of nanoparticle clusters: (**a**,**b**) ZnO, (**c**,**d**) TiO_2_, and (**e**,**f**) zeolite.

Furthermore, characterization of nanoparticles distributions by EDX mappings were made and the results are illustrated in Figure 3 and Figure S1 (from the Supplementary Material File). These SEM and EDX analyses revealed the proportions of particles clusters (associations), their morphology, and also their composition.

**Figure 4:**
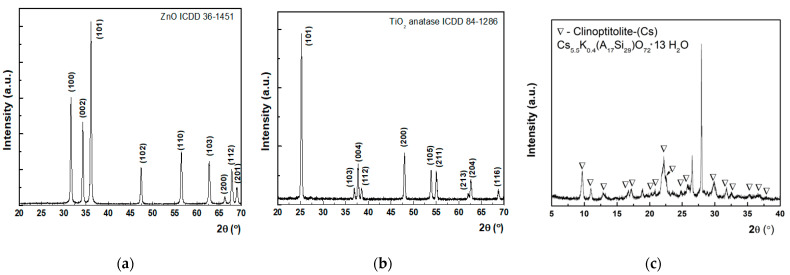
XRD patterns of the synthesized nanoparticles: (**a**) ZnO, (**b**) TiO_2_, and (**c**) zeolite.

XRD patterns of the synthesized nanoparticles are depicted in Figure 4. These graphs were obtained by employing the PXRD technique (powder X-ray diffraction), which is a rapid technique that measures the diffraction pattern of crystalline material.

**Figure 5:**
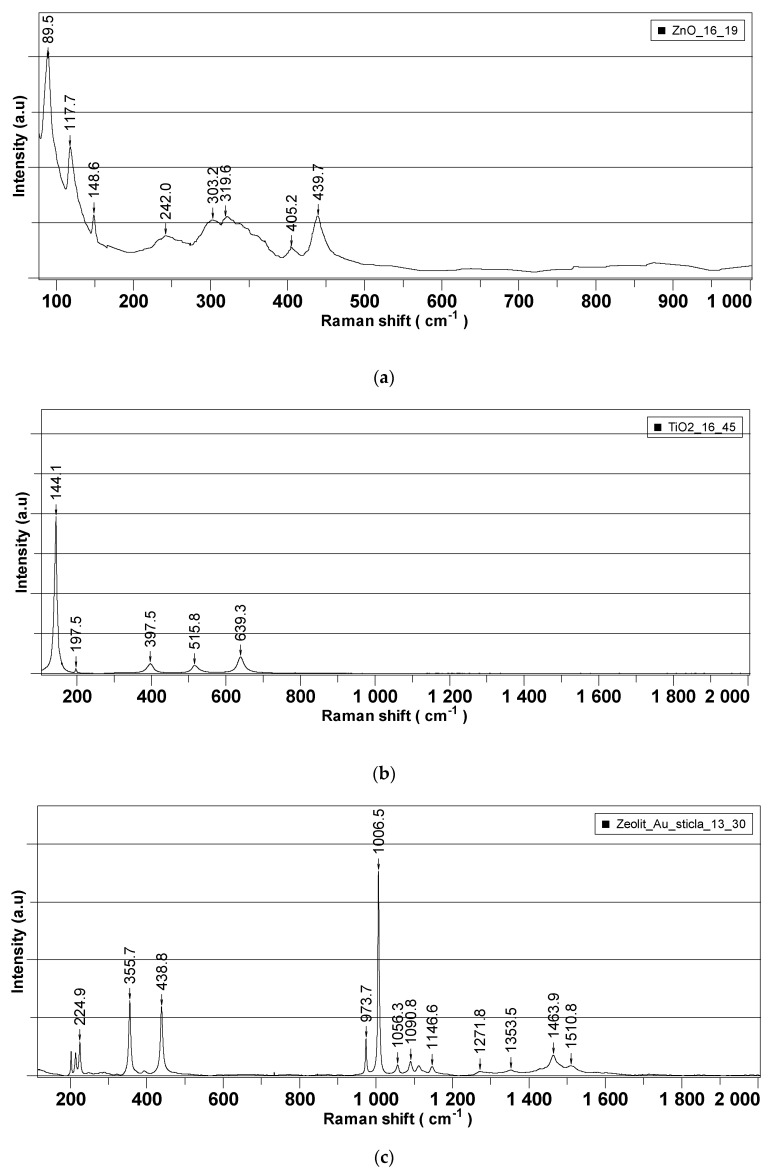
RAMAN spectra of the synthesized nanoparticles: (**a**) ZnO, (**b**) TiO_2_, and (**c**) zeolite.

**Figure 6:**
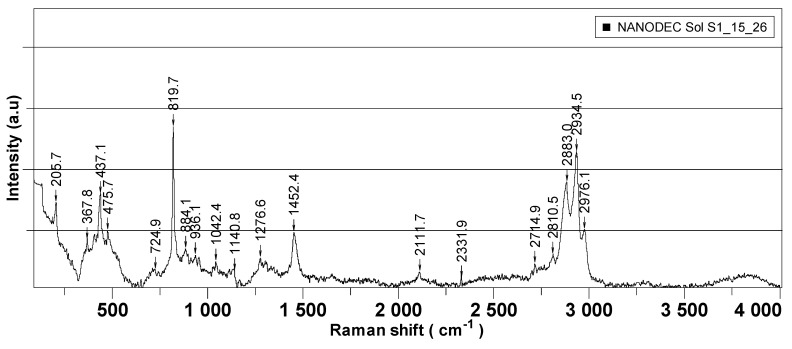
RAMAN spectra of the neat organic decontamination solution.

Figure 5 presents the Raman spectra recorded for each of the synthesized nanoparticles, which were further employed as active components in the decontamination solutions. Figure 6 is comprised of the Raman spectra of the neat organic decontamination solution.

After obtaining the decontamination solutions, their efficiency against real and simulated biological and chemical warfare agents was evaluated through specific procedures, as detailed in the Methods section. Initially, an extensive evaluation of the antimicrobial activity of the neat organic decontamination solution was performed, followed by a comparative evaluation of the neutralization efficacy of the NPs-based solutions. These tests were performed on real biological warfare agents (e.g., *Bacillus anthracis*) and also on other microorganisms which can be considered as simulants for various types of biological agents. Firstly, a controlled contamination was performed and the values obtained are presented in Table 2. Afterwards, the decontamination solution was applied on the contaminated samples. The results obtained after the completion of the decontamination process are illustrated in Table 3.

After the evaluation of the neat organic solutions, the NPs-enriched decontamination solutions were also subjected to analysis for establishing their influence on the biological decontamination capacity.

It is worth mentioning that the solutions comprising the nanoparticles were firstly subjected to the same procedures as the neat DS but we obtained almost similar results probably due to the particularities of each surface tested (porosity, rugosity, etc.), which have a major influence on the success of the decontamination process, and probably also due to the too low concentration of NPs employed for these tests. Thus, we can affirm that we were not able to prove the contribution of the NPs to the improvement of the decontamination efficiency of DS through the classical methods presented in NATO standards. Therefore, we employed other procedures for biological and chemical decontamination, which implied direct contact between the contaminants and the decontamination suspensions comprising the NPs, as further described.

Given the presence of nanoparticles in these new decontamination solutions (nanoparticles in suspension), other microbiology techniques, as detailed below, were better suited for the evaluation of their antimicrobial activity. Therefore, for this purpose, the minimal inhibitory concentration (MIC), minimal bactericidal concentration (MBC), and time-kill test methods were employed for comparing the decontamination performances of NPs’ suspensions (Table 4).

The time kill assay (Figure S3—Supplementary Material File) was performed only on *E. coli and S. aureus*, and the suspensions used to perform the tests were 2 × 10^7^ CFU/mL for *E. coli* and 6 × 10^7^ CFU/mL for *S. aureus*. The plates inoculated after 3 h of contact had sporadic growth for the DS-ZnO-0.5 and DS-ZnO-1 samples on each microorganism. After 6 h of contact, no signs of growth were observed for any microorganism. The number of CFU recovered after the incubation time were between 1 and 15 CFU/MHa plate for *Ecoli* and between 3 and 25 CFU/MHa plate for *S. aureus.* The results were, even between replicates, very different. Unfortunately, the low level of CFU/agar plates (maximum 25 CFU/plate) and the differences between replicates make these results unreliable for a statistical interpretation [36].

**Figure 7:**
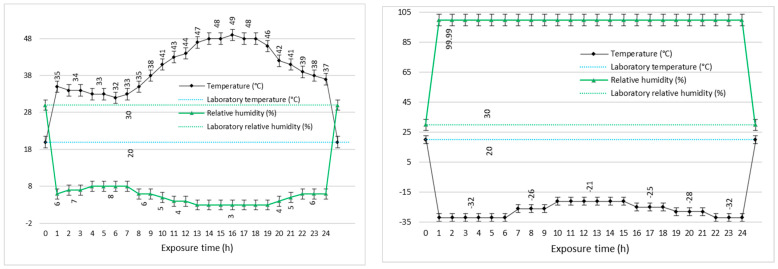
Temperature cycles assay on neat DS.

**Figure 8:**
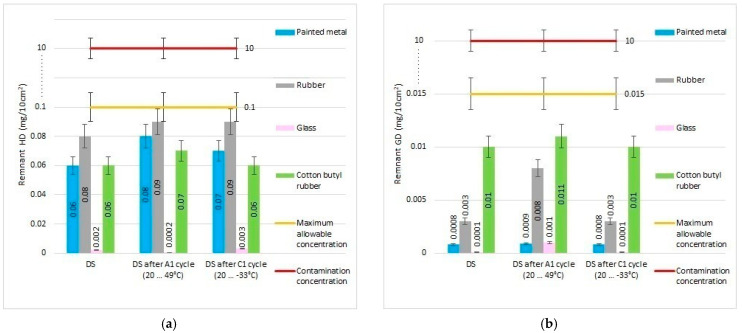
Remnant toxic concentrations (**a**) HD and (**b**) GD after decontamination with neat DS on different types of surfaces.

In parallel, an extensive evaluation of the decontamination efficacy for two real chemical warfare agents (yperite and soman) was performed, utilizing the neat organic solution along with the decontamination solutions enriched with the nanosized adsorbents. Prior to the dispersion of the nano adsorbents in the organic solution, the neat decontamination solution (sample DS, Table 1) was tested against chemical warfare agents (described in detail in the Methods section) before and after being subjected to various temperature cycles. These tests had the purpose of demonstrating that neat DS maintains its decontamination performances even after being subjected to extreme environmental conditions (Figure 7), this being an important feature for the formulations employed in military operational scenarios. The decontamination capacity of neat DS against sulfur mustard (HD) and soman (GD) on different types of surfaces was evaluated and the results are detailed in Table 5 while the remnant toxic concentrations are depicted in Figure 8.

**Figure 9:**
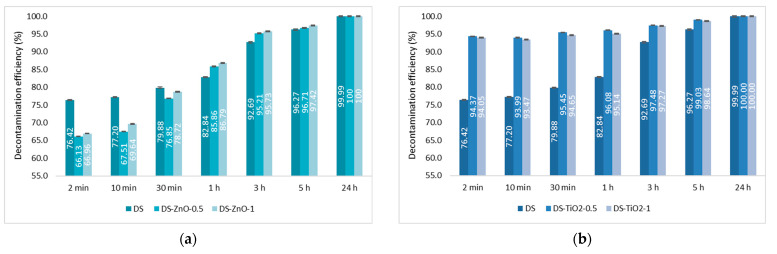

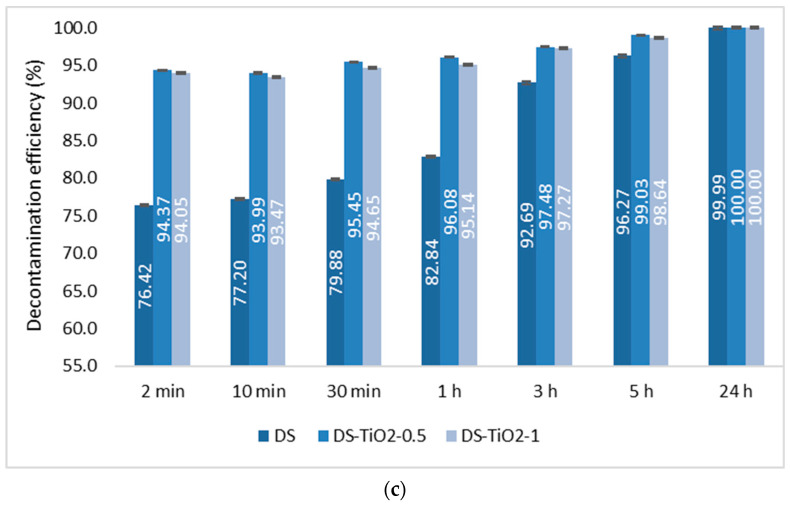
Decontamination degrees (%) of sulfur mustard (HD) obtained for decontamination formulations containing: (**a**) ZnO, (**b**) TiO_2_, and (**c**) zeolite (detailed statistical data presented in Tables S9 and S10—Supplementary Material File).

**Figure 10:**
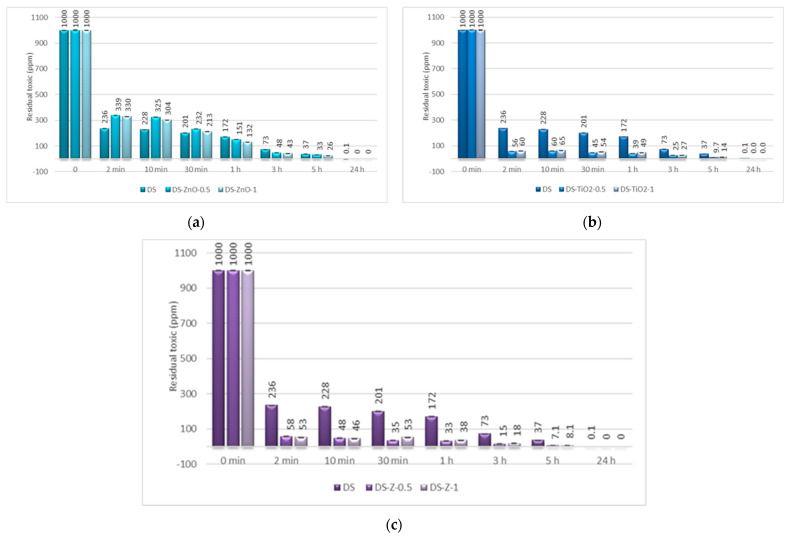
Residual sulfur mustard after employing decontamination formulations based on (**a**) ZnO, (**b**) TiO_2_, and (**c**) zeolite (detailed statistical data presented in Tables S9 and S10—Supplementary Material File).

**Figure 11:**
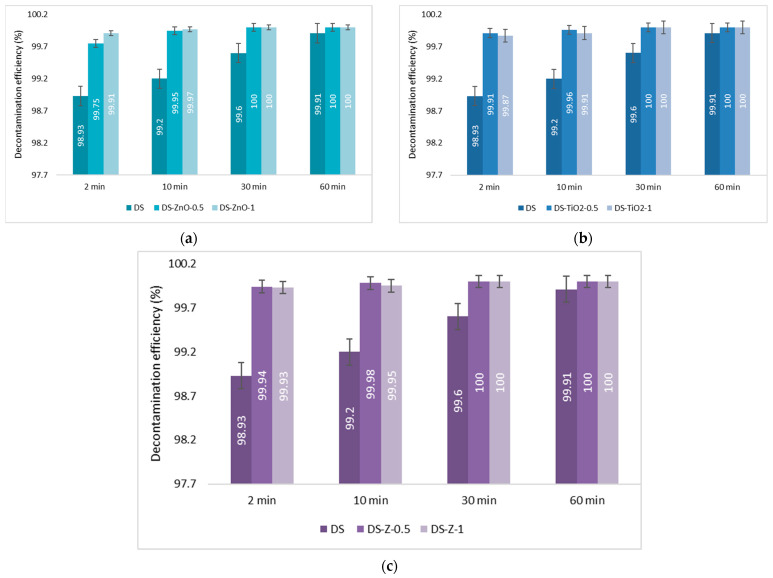
Decontamination degrees (%) of soman (GD) obtained for decontamination formulations containing: (**a**) ZnO, (**b**) TiO_2_, and (**c**) zeolite (detailed statistical data presented in Tables S9 and S10—Supplementary Material File).

**Figure 12:**
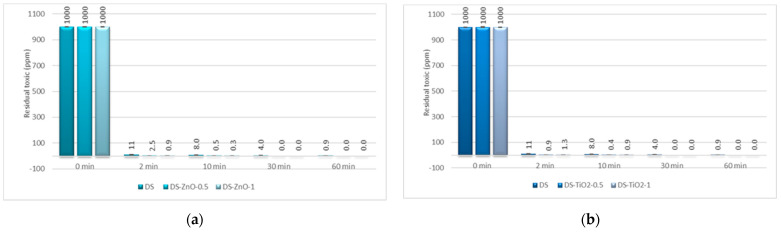

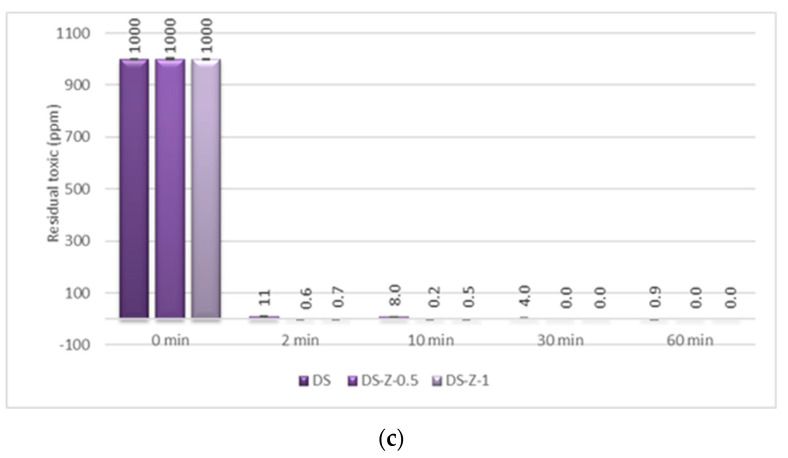
Residual soman after employing decontamination formulations based on (**a**) ZnO, (**b**) TiO_2_, and (**c**) zeolite (detailed statistical data presented in Tables S9 and S10—Supplementary Material File).

The last step consisted of the evaluation of the decontamination efficacy of the NPs-based decontamination solutions against HD and GD. The decontamination degrees obtained for decontamination formulations and the remnant toxic concentrations are summarized in Figure 9, Figure 10, Figure 11 and Figure 12. For all these experiments, the decontamination efficiency was calculated according to the formula described in the Methods Section, DF = 100∙(C_0_ − C_f_)/C_0_, thus the values obtained for the decontamination factor (DF) are all displayed in these figures in percentage units (%). DF represents the difference calculated between the initial concentration (time 0) and the concentration measured at a specific moment relative to the initial contamination (at time 0 min).

## 3. Discussion

The present work is comprised of a decontamination study on real and simulated biological and chemical warfare agents, in which the focus was on evaluating the antimicrobial activity and capacity of neutralizing the chemical warfare agents of three different types of organic solutions enriched with ZnO, TiO_2_, and zeolite nanoparticles specially designed for decontamination applications. The first step of this study consisted of the synthesis of the nanoparticles, followed by the development of the decontamination suspensions. The synthesis of ZnO nanoparticles, as detailed in the Methods section below, led to round–shaped nanoparticles (Figure 1a) with dimensions ranging in the 50–230 nm interval. These nanoparticles were further employed for obtaining the decontamination suspensions DS-ZnO-0.5 (containing 0.5 wt. % ZnO NPs) and DS-ZnO-1 (containing 1 wt. % ZnO NPs). The morphology of TiO_2_ nanoparticles was similar with the one of ZnO nanoparticles. Round–shaped TiO_2_ (Figure 1b) nanoparticles, most of them measuring between 50 and 210 nm, were employed for the synthesis of the DS-TiO_2_-0.5 (containing 0.5 wt. % TiO_2_ NPs) and DS-TiO_2_-1 decontamination suspensions (containing 1% TiO_2_ NPs). The micronized zeolite clinoptilolite (MZC) was subjected to ultrasonication in isopropyl alcohol prior to its addition in the decontamination solution in order to reduce the size of the particles for ensuring a higher active specific surface. SEM images (Figure 1c) revealed the morphology of the zeolite nanoparticles obtained through the above-mentioned procedure. As can be observed, zeolite particles do not possess a regular shape, with both isolated nanoparticles (ranging from 50 to 350 nm, Figure 2c) and mesoporous aggregates being present. Even if the dimensions of zeolite nanoparticles were higher than the ones of ZnO and TiO_2_, their high porosity ensured an efficient decontamination capacity of the suspensions in which they were employed, namely DS-Z-0.5 (containing 0.5% zeolite NPs) and DS-Z-1 (containing 1 wt. % zeolite NPs). SEM−EDX investigations revealed the proportions of particle clusters (associations), their morphology, and also their composition. EDX mapping (Figure 3d–f) offered evidence on the distribution of each identified element and also on their relative abundance. As can be observed in Figure 3, in comparison with ZnO and TiO_2_, which displayed a homogeneous distribution of the constituent elements, the atoms identified in the zeolite displayed a disparate distribution.

Powder X-ray diffraction investigations were performed on all the three types of nanosized adsorbents which were further introduced in the decontamination formulations. Thus, PXRD patterns of the investigated materials, namely (a) ZnO, (b) TiO_2_, (c) zeolite, can be examined from Figure 4. As can be observed, each pattern presents multiple diffraction peaks further assigned according to the ICDD (International Centre for Diffraction Data) database. The XRD pattern for ZnO powder, as shown in Figure 4a, showed the presence of pure wurtzite ZnO with the crystalline lattice constant, namely a = b = 3.25 Å and c = 5.22 Å, belonging to the P63(mc) space group, in accordance with the standard database (JCPDS file 36-1451). The mean crystallite size was evaluated as 33.1 nm (Scherrer equation [38]) and with a tensile lattice strain of 0.12%. No peaks corresponding to impurities were detected, showing that the final synthesis products purely consist of ZnO. Similarly, for TiO_2_ powder, the presence of pure anatase phase was shown with lattice constants a = b = 0.37 nm and c = 0.95 nm, belonging to the I41/amd tetragonal space group, consistent with the standard database (JCPDS file 84-1286). The mean crystallite size was evaluated as 32 nm (Scherrer equation) and with a tensile lattice strain of 0.10%. No peaks corresponding to impurities were detected, showing that the final products purely consist of TiO_2_ anatase phase. In the case of zeolite, it seems that a phase combination is involved. As it resulted from the XRD spectra after phase identification, it belongs to the heulandite group, which may include several clinoptilolite specimens. Regarding the investigated zeolite, specifically clinoptilolite (Cs_5.5_K_0.4_ (Al_7_Si_29_) O_72_H_2_O), used in this paper, it seems that a phase combination is involved. According to the phase identification, clinoptitolite (Cs_5.5_K_0.4_ (Al_7_Si_29_) O_72_H_2_O) was found alongside the monoclinic phase with a = 1.77 nm, b = 1.79 nm, and c = 0.74 nm, while the angles had the following values: α = 90°, β = 116.18°, and γ = 90°. The unidentified diffraction peaks might be assigned to laumontite monoclinic phase (Ca_7_ Al_14_ Si_26_ O) or muscovite (H_2_ K Al_3_ Si_3_ O_12_). The good crystalline quality in each case is confirmed by the mean crystallite sizes above 20 nm according to Scherrer’s equation [38].

Raman analysis was performed separately on nanoparticles’ powders and on the neat DS, and the results are illustrated in Figure 5 and Figure 6, respectively. The properties of nanosized materials are strongly related to their structure; therefore, the structure of the synthesized nanoparticles was studied to understand the structure–property relationships in correlation with the application for which the nanoparticles were designed for. Raman spectra provided supplementary information about the crystalline structure of the investigated samples. According to the group theory, the wurtzite ZnO single-crystal belonging to the 4 6 v C (P63mc) space group has 1A1, 2B1, 1E1, and 2E2 optical phonon modes [39,40]. The non-polar phonon mode, namely E2, had two frequencies: the E2 (low) and E2 (high) modes, which are associated with the non-polar vibration of the heavier Zn atoms sublattice and of the lighter oxygen atoms sublattice, respectively. Figure 2 shows the Raman spectra in the range of 70–1000 cm^−1^ for the prepared ZnO nanoparticles. The Raman spectra of all the samples exhibited the E2 (high) and E2 (low) modes at ~439.6 cm^−1^ and ~89.4 cm^−1^, respectively, demonstrating that ZnO-prepared NPs’ structures are of wurtzite hexagonal phase. The peaks corresponding to E2 (high) and E2 (low) have a reasonable line width of about 15 cm^−1^ for all the measured samples and no broad Raman signal in the range of 500–1000 cm^−1^ was found, which confirms the crystal quality of the prepared nanoparticles. At the same time, the peaks corresponding to the E2 (high) mode are red-shifted for all the samples from ~439.6 cm^−1^ (437 cm^−1^ for ZnO bulk crystal) most likely due to the presence of some impurities (less than 0.5%) and/or a small strain in the structures. These Raman shifts of the main peaks existing in the Raman spectra of ZnO NPs are generally characteristic for ZnO-prepared/synthesized nanostructures, with different morphologies and defects. Titanium dioxide (TiO_2_) is a polymorphic material occurring in three naturally crystalline phases: anatase, rutile, and brookite [41]. TiO_2_ is also an environmentally friendly material with various contents of these polymorphs, which have been attracting great interest because of their mechanical, electrical, optical, biological, and magnetic properties [42]. Anatase phase is tetragonal, with two formula units per unit cell, and has six Raman active modes (A1g, 2B1g, and 3Eg); these Raman vibrational modes are centered at 144 cm^−1^(E_g_), 197 cm^−1^ (E_g_), 399 cm^−1^(B1_g_), 513 cm^−1^(A1_g_), 519 cm^−1^(B1_g_), and 639 cm^−1^(E_g_). According to the recorded spectra (Figure 5b), the position of the Raman peaks (except A1g and B1g that cannot be visible individually at room temperature) are characteristic to the anatase phase of TiO_2_ nanoparticles. The zeolite analyzed by Raman spectroscopy comes from Romania, extracted from the Transylvanian region (specifications detailed in the Materials section). Despite the existence of a significant number of research papers dealing with the Raman spectra of zeolites, the clear assignment of the peaks that appear in the spectrum remains a challenge. A method of analyzing the Raman spectrum for a zeolite can be done taking into account the Si/Al ratio which can vary by replacing the Al atoms with Si atoms [43] . Thus, in our case where the ratio is greater than 4, the peaks that appear are positioned at 355,7 cm^−1^, 438.8 cm^−1^, 973.7 cm^−1^, 1006.5 cm^−1^, 1058.3 cm^−1^, or 1090 cm^−1^, 1146.7 and are due to Si-O-Al and Si-O-Si vibrations. Raman peaks in the region of 1200–1510 cm^−1^ can be attributed to the contamination of the analyzed sample with organic compounds. For neat DS, Raman peaks in the interval of 367–475 cm^−1^ could be assigned to C−C vibrations. The peak found at 205 cm^−1^ appeared due to the presence of NaOH in the decontamination solution. In the region of 800–1150 cm^−1^, the specific peaks for C−O−C bonds can be observed. The presence of isopropyl alcohol in the neat DS generated the two peaks found at 819 cm^−1^ (C-H) and 1452 cm^−1^ (OCH bending). The peaks ranging from 2800 to 2970 cm^−1^ are specific for C-H vibrations.

### 3.1. Biological Decontamination

It was found that on the sporulated forms of *B. anthracis* and also on its vegetative form, the decontamination solution showed excellent antimicrobial activity, according to Table 3. In the case of *Ps. aeruginosa* and the vegetative form of *B. cereus* and *B. subtilis*, we observed no logarithmic reduction on the painted metal sample, the microorganisms being too many to be counted. For *S. aureus* in vegetative form, regarding the results on all four types of surfaces, a reduction with log 3 was shown, while for *B. cereus*, *B. subtilis*, and *P. aeruginosa*, a logarithmic reduction of 3 was noticed in the case of glass, rubber, and cotton butyl rubber surfaces. A substance is considered to have bactericidal effect if at least a 3Log reduction in CFU/mL or 99.9% kill is obtained over a specified time (24 h) of contact. Bactericidal effect, in some cases, can be determined at 6 h. A 90% kill (1Log reduction) at 6 h is the equivalent to a 99.9% reduction at 24 h [44]. *B. cereus* and *B. subtilis* in the sporulated form presented a reduction of log 2 on painted metal and cotton butyl rubber, and a reduction of log 1 for glass and rubber. For the sporulated forms, the reduction should be between 1 and 1.5 log [45] to be considered decontaminated.

All the studied suspensions demonstrated antimicrobial activity against the tested microorganisms. The MIC values of all the substances are in the range of a maximum two-fold dilution, which is quite homogeneous. This homogeneity of the MIC’s, most likely, is due to the same way of the action of the substances. The most sensitive microorganism to the decontamination solutions seems to be *Bacillus spizizenii*. In the case of *Ps. aeruginosa*, the MIC values are all the same, but the MBC values are the most diverse. The time kill assay was performed only on *E. coli and S. aureus*. In the test condition, no signs of growth were observed for any of the microorganisms put in contact with DS, DS-TiO_2_-0.5, DS-TiO_2_-1, DS-Z-0.5, and DS-Z-1 even after 3 h, which means an almost 6Log decrease. In the case of the nanoparticles solutions based on DS-ZnO-0.5 and DS-ZnO-1 only after 6 h of contact, the same decrease was observed for both studied microorganisms. According to Konate et al. l, a 1Log decrease after 6 h of contact demonstrates the bactericidal effect of a substance with antimicrobial properties. When comparing the MBC and time-kill test, the results may seem different, but we must take into account that in the case of MBC, microorganisms were in contact with nanoparticles in MHb, a nutrient medium, and in the case of time-kill test, the microorganisms were in contact in PBS.

### 3.2. Chemical Decontamination

The decontamination tests on different surfaces represent a very useful tool to evaluate, at a small scale, an operational decontamination process. The testing method combines the requirements for the laboratory procedures with the operational ones. The samples tested represent the most common surfaces that are found near a military station: painted metal (technicals, cars, and armament), glass (goggles for a gas mask, sniper scope, and windows), rubber (protective gloves, gas mask, and gaskets), and cotton butyl rubber (individual protective equipment).

The remnant toxic chemical concentration was evaluated by GC-MS on the neat DS and on the neat DS after being exposed to a hot-dry cycle temperature and after a cold cycle temperature. The results showed a decontamination efficiency for HD between 99.2% and 99.98% for the neat DS after 10 min. In comparing the results of DS and DS after extreme temperature exposure, we observed a slightly increment of the remnant concentration on the surfaces, but the decontamination efficiency was still over 99% on all the surfaces with all three tested solutions, and a minimum decontamination efficiency for blister agents is required by NATO standards (Table 5). In comparing the different types of tested surfaces, we observed the expected fact that the remnant HD on porous materials such as rubber (0.09 mg/10 cm^2^) is higher than for surfaces such as glass (0.0002 mg/10 cm^2^) from an initial contamination of 10 mg/10 cm^2^ (Figure 8a). The GD decontamination on different surfaces showed superior results of about 99.93% (rubber) to 100% (glass) (Table 5). A slightly higher concentration of remnant GD was observed on cotton butyl rubber (0.01 mg/10 cm^2^; Figure 8b) but, even so, the decontamination efficiency is higher than the 99.90%, minimum decontamination efficiency required by NATO standards for nerve agents.

The procedure for decontamination on multiple surfaces implies a water rinse step, which helps the removal of the toxic chemical, a very important fact in operational decontamination. In order to evaluate the decontamination efficiency enhancement of the NPs’ addition, the procedure had been changed in order to strictly evaluate the degradation on the toxic chemical and not the degradation together with the removal, as we referred to in the first procedure.

The decontamination efficiency of the decontamination formulations based on ZnO, TiO_2_, and zeolite nanoparticles was evaluated after 2 min, 10 min, 30 min, 1 h, 5 h, and 24 h for sulfur mustard and after 2 min, 10 min, 30 min, and 1 h for soman (Figure 9, Figure 10, Figure 11 and Figure 12). Suspensions of 0.5 wt. % NPs and 1 wt. % NPs were tested for the decontamination efficiency.

For the decontamination of HD, we observed an improvement considering the decontamination efficiency and the remnant toxic on the samples containing NPs. The neat organic DS presented a decontamination efficiency of 82.84% after 1 h, equivalent to 172 ppm remnant HD, and of 96.27% after 5 h, equivalent to 37 ppm remnant HD. The results obtained from the decontamination with NPs’ suspensions showed an improvement of the reaction speed, with the decontamination efficiency after 1 h being 96.74% (DS-Z-0.5), 96.08% (DS-TiO_2_-0.5), and 86.79% (DS-ZnO-1; Figure 9). The equivalent of remnant HD after 1 h is between 33 ppm and 132 ppm (Figure 10). We can observe that the results obtained after 1 h with the NPs’ suspensions of TiO_2_ and zeolite are almost the same as after 5 h with the neat organic DS. After 5 h, the NPs’ suspensions showed a decontamination efficiency of over 99% (DS-TiO_2_-0.5, DS-Z-0.5, and DS-Z-1; Figure 9), equivalent to less than 10 ppm remnant HD, from an initial concentration of 1000 ppm HD (Figure 10).

In the case of GD decontamination, the neat organic DS presented a 98.93% decontamination efficiency after only 2 min (Figure 11), equivalent to 11 ppm (Figure 12), and 99.91% after 1 h, equivalent to less than 1 ppm. Even if, in the case of this nerve agent, the DS worked very fast and with a very good neutralization reaction, the NPs’ suspensions showed also a faster degradation with an improved decontamination efficiency. After 2 min, the suspensions showed decontamination efficiencies of about 99.91% (DS-ZnO-1 and DS-TiO_2_-0.5) and 99.4% (DS-Z-0.5; Figure 11), representing less than 1 ppm remnant GD (Figure 12). After 30 min, all six NPs’ suspensions tested presented a completed neutralization of the GD.

### 3.3. Statistical Analysis of the Decontamination Experiments Results

For a brief statistical evaluation of the decontamination efficiency of the solutions employed in this study, neat DS was compared with each of the nanoparticle-enriched formulations. For the decontamination efficiency of HD statistical analysis, DS was compared with each nanoparticle suspension. A threshold of significance of α = 0.05 was attributed for the validation of the statistical results and 0.95 confidence level. It was hypothesized (H_0_) that there is no significant statistical difference between the mean values of the concentrations of the two compared samples. For this purpose, we applied the paired T-student test. From the results’ analysis, in the case of the DS-ZnO–0.5 and DS-ZnO–1 comparison, the P(T ≤ t) two-tailed test statistics (0.014) offer the possibility to obtain the absolute t-value (3.268) and evaluated it as being higher than the critical t-value (2.364). As the statistical significance value (P) is lower than the threshold of significance (α) originally set, the initial hypothesis H_0_ is rejected in this case. Therefore, it results that there is a significant difference between the mean concentration values of each specimen, thus $\overset{-}{x}$ DS-ZnO–0.5 = 265.921 and $\overset{-}{x}$ DS-ZnO–1 = 255.919. In the case of the DS and DS-ZnO–0.5 comparison, the absolute t-value (−1.260) is lower than the critical t-value (2.364). The values of the statistical significance are higher than the threshold of significance α, therefore the initial hypothesis is accepted and it can be concluded that there are no significant differences between the mean HD remanent concentrations of each sample. Table S4 from the Supplementary Material File provides the information representing that the DS-ZnO–0.5 and DS-ZnO–1 in comparison with DS are not significantly different as the mode of action on the HD and the remnant concentration of the toxicant are similar. In the case of the DS and TiO_2_ nanoparticle formulations, all three sets of comparisons showed a statistical significance lower than the threshold of significance initially set and the H_0_ hypothesis was rejected, leading to the conclusion that there is a significant difference between the measured samples. In the case of the DS and zeolite nanoparticle formulations, comparisons showed a P lower than α, leading to a rejected H_0_ hypothesis, but the comparison of the two different zeolite formulations showed a P higher than α, which is leading to the acceptance of the H_0_ hypothesis. As a conclusion, even if there is a significant difference between the HD remnant mean concentrations of the neat DS and each zeolite formulation, the two different concentrations of zeolite formulations showed no significant difference between them. The statistical analysis for the decontamination efficiency of GD (presented in Table S9 from the Supplementary Material File) showed, in all cases, no significant statistical difference between the mean values of the concentrations of each of the two compared samples, with the aspect that each of the two nanoparticle formulations (with the same type of nanoparticles) showed a more obvious resemblance than when they were compared to neat DS.

## 4. Materials and Methods

### 4.1. Materials

The neat DS was provided by the Research and Innovation Center for CBRN Defense and Ecology, 225 Oltenitei Ave., Bucharest, 041327, Romania. The materials they employed for the preparation of the neat decontamination solution were 2-ethoxyethanol (≥99.8%, ethylene glycol monoethyl ether, Sigma Aldrich, St. Louis, USA), monoethanolamine (≥98%, Sigma Aldrich, St. Louis, MO, USA), sodium hydroxide (≥98%, Sigma Aldrich, St. Louis, USA), isopropyl alcohol (≥99.7%, Sigma Aldrich, St. Louis, USA), and sodium lauryl sulfate (SDS, Sigma Aldrich, St. Louis, USA), and were used as received. Nanosized adsorbent ZnO was synthesized from zinc nitrate hexahydrate (Zn(NO_3_)_2_ × 6H_2_O, ≥98%, Sigma Aldrich, St. Louis, USA) and sodium hydroxide (≥97%, Sigma Aldrich, St. Louis, USA). Titanium dioxide anatase phase nanoparticles were synthesized from tetrabutyl titanate (97%, Sigma Aldrich, St. Louis, USA) and ethanol (99.8%, Sigma Aldrich, St. Louis, USA). The micronized zeolite clinoptilolite (MZC), with an average diameter of the particles of 5 microns, was purchased from Zeolites Production S.A (Cluj-Napoca, Romania), from Rupea Zeolite Mines, located in the central region of Romania. The zeolite was subjected to sonication in isopropyl alcohol (≥99.5%, Sigma Aldrich, St. Louis, USA). For the biological tests, the following biological real agents and simulants were used: *Bacillus anthracis* spores, *Bacillus cereus* spores, *Bacillus subtilis* (*spizizenii*) spores (ATCC 6633), *Bacillus anthracis*, *Bacillus cereus*, *Bacillus subtilis*, *Staphylococcus aureus* (ATCC 6538), *Pseudomonas aeruginosa* (ATCC 9027), and *Escherichia coli* (ATCC 8739). For the decontamination tests, real chemical warfare agents were utilized, including: Bis(2-chloroethyl) sulfide (HD, sulfur mustard, purity: 95%, CAS: 505-60-2, Schedule 1A(4) in the Chemical Weapons Convention (CWC), own synthesis) and soman (GD, pinacolyl methylfluorophosphonate, purity 90%, CAS: 96-64-0, Schedule 1A(1) in the CWC, own synthesis). The samples’ preparation for the GC-MS analyses involved di-chloromethane (≥ 99.8%, DCM, Merck Millipore, Kenilworth, NJ, USA). All the tests involving the toxic agents utilized in this study were performed at the Research and Innovation Center for CBRN Defense and Ecology, in the ‘Chemical Analysis Laboratory’, which is an OPCW Designated Laboratory from Romania.

### 4.2. Methods

#### 4.2.1. Synthesis of Nanosized Adsorbents for BCWA Decontamination Formulations

Zinc oxide nanoparticles

Zinc oxide nanoparticles were synthesized by using zinc nitrate hexahydrate ((Zn(NO_3_)_2_ × 6H_2_O) and sodium hydroxide as precursors. All the reagents (analytical grade purity) were purchased from Sigma Aldrich and used as received without any further purification. Zinc nitrate hexahydrate (Zn(NO_3_)_2_ × 6H_2_O (3.05 g)) was added into 100 mL of deionized water at room temperature and 0.8 g of NaOH was added in 200 mL of deionized water. NaOH solution was slowly dropped into the vigorously stirred Zn(NO_3_)_2_ solution for 1 h. A white precipitate was generated at the end of the reaction, which was collected by filtration (PTFE membrane, pores diameter of ≤ 0.5 µm). The particles obtained were washed with water and ethanol several times and dried at 80 °C for 24 h. ZnO nanostructures were obtained by annealing the as-prepared precursor at 250 °C for 2 h in a tube furnace.

Titanium dioxide nanoparticles

Titanium dioxide anatase phase nanoparticles were synthesized by chemical route from solution. The raw materials from which we started were tetrabutyl titanate (Ti(OCH_2_CH_2_CH_2_CH_3_)_4_, 97%) and ethanol (CH_3_CH_2_OH), purchased from Sigma-Aldrich. For synthesis of anatase TiO_2_ nanoparticles, an ethanol solution of tetrabutyl titanate was prepared (20 mL) and stirred for 30 min. Tetrabutyl titanate ethanolic solution was slowly added into 20 mL of deionized water, vigorously stirred, and then ultrasonicated for 15 min. The resulting precipitate was washed several times in deionized water and maintained in vacuum at room temperature for 24 h for the evaporation of solvent traces. Finally, the obtained titanium dioxide nanoparticles were annealed in air at 500 °C for 1 h.

Zeolite nanoparticles

The micronized zeolite clinoptilolite (MZC) [46,47], consisting of particles with an average diameter of 5 microns, was subjected to sonication in isopropyl alcohol for 3 h for the disaggregation of zeolite into smaller particles in order to enhance the total active specific surface and convert it into a nanosized adsorbent. For SEM analysis, after sonication, the alcoholic dispersions were deposited by dripping on silica support and dried in an oven at 70 °C. SEM analysis confirmed the accomplishment of this objective of turning zeolite into smaller nanometric particles.

#### 4.2.2. Biological Decontamination Tests

To test the antimicrobial effect of the decontamination solutions, several specific methods and techniques were used corresponding to the complexity of the microbiological trials, characteristics of the decontamination formulations, and type of surface subjected to decontamination. All the microbiology assays were accomplished by following NATO standards (AEP 58 [37] and AEP 7 [48]), adapting the methods for testing polyvalent decontaminants for surfaces. Neat DS solution was tested on four different types of surfaces (metal, glass, rubber, and cotton butyl rubber) against *Bacillus anthracis, Bacillus cereus, Bacillus subtilis, Staphylococcus aureus,* and *Pseudomonas aeruginosa.* For these tests, a controlled contamination with the above enumerated microorganisms was priorly performed for each type of surface and evaluated as follows: the initial microbial load was 10^4^ CFU/10 cm^2^ for both spores and vegetative forms. Contamination control was performed by employing pre-moistened sterile swabs for sampling the microorganisms from the contaminated areas (measuring 10 cm^2^). The collected samples were allowed to grow on solid culture media. To verify contamination, colony-forming units (CFU/cm^2^) were counted from the surface of the solid culture media. The values counted are illustrated in Table 2. After quantifying the initial contamination, we proceeded to the decontamination step. For this purpose, several steps were done, including: pre-washing with water (3 mL/10 cm^2^), applying decontamination solution (neat DS, 0.5 mL/10 cm^2^), allowing for the decontamination formulation to neutralize the targeted biological agents (10 min), and post-washing with water (3 mL/10 cm^2^). Decontamination control was performed by employing pre-moistened sterile swabs for sampling the microorganisms from the decontaminated areas (measuring 10 cm^2^). The collected samples were allowed to grow on solid culture media. To verify the decontamination efficiency, colony-forming units (CFU/cm^2^) were counted from the surface of the solid culture media. The values obtained at the completion of the decontamination are summarized in Table 3. Triplicate experiments were performed and the mean values are reported.

After the assessment of the antimicrobial activity of the neat organic solutions, the NPs-enriched decontamination solutions were also subjected to analysis for evaluating their influence on the biological decontamination capacity. Due to the presence of nanoparticles in these new decontamination solutions (nanoparticles in suspension), the minimal inhibitory concentration (MIC), minimal bactericidal concentration (MBC), and time-kill test, as detailed below, were better suited methods for the evaluation of their antimicrobial activity. The complementary assays MIC and MBC were employed for comparing the decontamination performances of NPs’ suspensions and the results are depicted in Table 4.

Minimal inhibitory concentration

The antimicrobial activity of the NPs-based decontamination solutions was evaluated against *Staphylococcus aureus* (ATCC 6538) and *Bacillus subtilis* subsp. *spizizenii* (ATCC 6633) as a model for Gram-positive bacteria, and *Escherichia coli* (ATCC 8739) and *Pseudomonas aeruginosa* (ATCC 9027) as a model for Gram-negative bacteria. *S. aureus*, *E. coli*, and *Ps. aeruginosa* were chosen, being considered standard microorganisms for testing the antimicrobial properties of newly synthesized products [49]. After cultivation overnight in tryptic soy broth (TSB; Merck) at 37 °C with stirring (200 rpm), the bacterial strains were harvested. Portions of the suspension were harvested by centrifugation and resuspended in phosphate buffer saline (PBS; Sigma-Aldrich). The suspensions were adjusted to approximately 10^6^ CFU/mL [50]. Minimum inhibitory concentrations (MIC) were established for each decontamination formulation by the broth microdilution method [51,52]. Two-fold serial dilution in Mueller-Hinton broth (MHb) of each composite were performed in duplicates. Negative and positive controls were associated [53]. The inhibitory effect of the substances was evaluated starting from 50% concentration (the samples of the substances were diluted at 1:1 with MHb). After diluting the sample, in each well, we added 10 µL of the microorganism suspensions (~10^4^ CFU). The same volume of the suspensions was added in the positive control wells.

Minimal bactericidal concentration

Minimal bactericidal concentration (MBC) values were also established. After the incubation period, we needed to establish MIC, specifically the content of the wells, which did not show that any visible signs of growth were plated on the appropriate agar plates. Agar plates were incubated for 48 h in order to reveal any surviving bacteria [53,54].

Time-kill test

A portion of more concentrated bacterial strain suspensions (10^7^ CFU/mL), prepared as previously described, were treated with the studied formulations at a final concentration of 2× MIC values and incubated at 37 °C with continuous shaking for 3 h, 6 h, and 24 h. At each established time, a portion of the bacterial cultures were serial-diluted in PBS and then plated in duplicates on Mueller-Hinton agar (MHa) medium. After incubation at 37 °C for 24 h, the bacterial survival was evaluated.

MIC, MBC, and time -kill tests were repeated four times for assessing the reproducibility of these experiments.

#### 4.2.3. Chemical Decontamination Tests

Extreme temperature exposure

The decontamination neat solution was exposed to extreme temperature cycles, specifically for 24 h for each cycle, in a climatic chamber in order to test the decontamination efficiency after this exposure. Category A1 (extreme hot–dry), which is the first 24 h cycle, applies to areas which experience very high temperatures, namely the hot–dry deserts of North Africa, parts of the Middle East, northern India, and south-western USA. Romania belongs to the A3 (intermediate hot) area but because the Romanian army carries out military actions in areas corresponding to A1, the solutions were exposed to the corresponding extreme temperature cycle (20 … 49 °C) and associated humidity (3% ... 8% relative humidity). Category C1 (intermediate cold), which is the second 24 h cycle (20 … −33 °C), applies to those areas that experience moderately low temperatures, such as central Europe, Japan, and the central USA (AECTP-230). At the end of each temperature cycle, the efficiency of the chemical decontamination of sulfur mustard and soman on samples of different materials was tested. 

Decontamination efficiency of DS on different types of surfaces

Decontamination efficiency tests were performed following the same procedure on the same types of materials (painted metal, glass, rubber, and cotton butyl rubber) using three decontamination solutions: neat DS, neat DS after A1 cycle exposure, and neat DS after C1 cycle exposure.

Contamination of samples with HD/GD. A 10 cm^2^ sample for testing (painted metal, glass, rubber, and cotton butyl rubber) was placed in a glass cuvette and contaminated using a micropipette with 8.29 µL 95% HD and 10.88 µL GD 90%, equivalent to 10 mg of pure toxic. The toxic chemical was distributed on the surface of the sample as droplets of about 0.7 to 1 µL each.

Decontamination of samples. After 30 min of contact time between the sample for testing and the toxic chemical, 0.5 mL of DS was pipetted from the edges to the center in continuous and thin film so that the surface was completely covered with SD. After 5 min of direct contact with toxic–SD, the sample was rinsed with 3 mL of water.

Sampling and preparation of samples for analysis. The sample was placed into a clean glass cuvette and extracted with 10 mL of dichloromethane (DCM) for 10 min under continuous stirring to ensure the best possible extraction of the remaining toxic agent. One mL of organic extract from the cuvette was sampled for chromatographic analysis to determine the amount of remaining toxic agent based on the calibration curve. A 5-point calibration curve was performed for each toxic, HD and GD, based on the concentration, at which the contamination was performed and also based on the maximum admitted concentration. The remnant concentration of the HD and GD after decontamination was calculated, according to AEP 58 [37].

Decontamination efficiency of NPs’ suspensions

Representative NPs’ suspensions and neat DS were tested for the decontamination efficiency by being put in direct contact as follows: three types of NPs (ZnO, TiO_2_, and zeolite) were used to prepare suspensions in 2 concentrations of 0.5 wt.% NPs and 1 wt. % NPs. The six suspensions together with neat SD were used to decontaminate HD and GD, and the decontamination reaction was monitored and quantified after several times until the reaction was completed and the toxic chemical was completely neutralized. Five mL from each suspension and the neat DS were contaminated with 5.25 µL of HD and 5.55 µL GD, respectively (1000 ppm of pure toxic in suspension). Decontamination took place at room temperature under magnetic stirring. In total, 200 µL of each suspension was extracted with DCM in order to be analyzed by GC-MS.

In all the decontamination experiments for evaluating the decontamination efficiency (thus calculating the decontamination factor (DF)), the equation employed was DF = 100∙(C_0_ − C_f_)/C_0_, where DF is the decontamination factor, C_0_ is the initial concentration of the contaminant, and C_f_ is the final concentration of the contaminant (indicating the residual contamination).

#### 4.2.4. Statistical Analysis

The decontamination experiments were run in triplicates (sample size = 3) and the results are expressed as mean standard deviation (as presented in Tables S1–S3 and Tables S6–S8 from the Supplementary Material File). Statistical analysis was performed using Microsoft Excel–Data analysis tools and Origin Lab–Data analysis tools. For this purpose, the statistical evaluation of the results was assessed with the t-test-paired two samples for means, which provides a hypothesis test of the difference between population means for a pair of random samples whose differences are approximately normally distributed. The paired T-student test method was chosen due to the small size sample (less than 30). A statistical significance of α = 0.05 was attributed for the validation of the statistical results and 0.95 confidence level.

### 4.3. Characterization

The morphology and size of the synthesized nanoparticles were observed by a SEM-Field Emission Scanning Electron Microscope (FEG-SEM)-Nova NanoSEM 630. The characterization was performed at magnifications of 120 kx, 100 kx, and 60 kx, and at acceleration voltages of 10 kV and 15 kV using the In-lens Secondary Electrons detector (TLD-SE). EDX mapping was fulfilled through SEM-EDX analysis performed with the aid of a Zeiss Gemini 500 microscope coupled with an XFlash 6 EDX detector from Bruker. All data from the EDX were analyzed using the ESPRIT 2 Software. Raman measurements were performed at room temperature with a Horiba micro-Raman spectrometer LabRAM HR 800, heliu-Neon Laser, which was 633 nm with a confocal Olympus microscope. A 100× objectiv was used to focus on ZnO nanoparticles’ powder and Raman-scattered light was collected through the same microscope objective and detected by a cooled CCD detector. For TiO_2_ nanoparticles, the excitation source was a red laser (λ = 633 nm) with an output power of 15 mW and it was focused to a spot of ~ 0.8 µm. Before measurement, the system was calibrated using the 521 cm-1 Raman line of a silicon (Si) wafer. Raman spectra for zeolite was collected with the powder placed on an Au/glass substrate in order to enhance Raman-scattered light. The light source was a He-Ne laser (632.8 nm), with the laser spot at < 1 µm and ×100 objective of the Olympus confocal microscope. X-ray diffraction investigations were performed using a 9 kW SmartLab diffractometer (Osaka, Japan) operated at 40 kW and 75 mA. Powder XRD (PXRD) patterns were recorded using a step of 0.01° with a speed of 5°/min in the θ/2θ configuration. Gas chromatography mass spectrometry analyses were performed on a GC Focus-MS DSQII, Thermo Electron Corporation (Waltham, United States). GC analyses were performed on a TR5MS capillary column, with the dimensions of 30 m × 0.25 mm × 0.25 μm, using ultra-pure Helium (6.0), a 1 mL/min constant flow, constant pressure, the spitless injection mode (15 mL/min), and an injection volume of 1 μL. The GC temperature program consisted of 60 °C (2 min) with a heating rate of 10 °C to 300 °C (10 min). MS analyses were performed under the electron impact (EI) ionization mode at 70 eV electron energy, with a 3 min solvent dela, on a scan range of 40–650 m/z. The extreme temperature cycles were performed into an Angelantoni Industrie SpA climatic chamber, specifically the DY110 model (Cimacolle, Italy).

## 5. Conclusions

This study focused on the investigation of the antimicrobial activity and degradation ability of three distinct types of nanoparticle-enriched formulations specially designed for the neutralization of real and simulated biological and real chemical warfare agents. The performances of these solutions were evaluated by simulating the operational conditions encountered on the battlefield or in a terrorist attack situation by investigating the efficiency of neutralizing real biological and chemical warfare agents from various types of surfaces (painted metal, glass, rubber, and cotton butyl rubber) that could be contaminated in the above-described scenarios.

The synthesized nanoparticles were characterized by means of SEM-EDX, XRD, and RAMAN techniques. The ZnO and TiO_2_ nanoparticles obtained displayed similar morphologies, having almost similar rounded shapes and comparable sizes. The shape of the micronized zeolite taken from Rupea (detailed in the Methods section) was reduced by ultrasonication in isopropyl alcohol and the mesoporous structure of the nanosized particles obtained was emphasized by SEM imaging. XRD offered evidence on the atomic structure of the crystals. The size, shape, and internal stress of the small crystalline regions were revealed. Raman analysis provided supplementary information about the crystalline structure of the investigated samples. Furthermore, these three nanosized adsorbents (ZnO, TiO_2_, and zeolite) were introduced in the neat organic solution DS, obtaining the corresponding decontamination formulations comprising 0.5 wt.% NPs and 1% NPs. These novel formulations were utilized for decontaminating biological agents (bacterial spores, Gram-positive bacilli, Gram-negative bacilli, and Gram-positive cocci) and chemical agents (sulfur mustard and soman). The biological decontamination efficiency was evaluated for both vegetative and sporulated forms of the tested microorganisms. The antimicrobial activity of the decontamination formulations were demonstrated by a logarithmic reduction of higher than three for the vegetative forms and higher than one for the sporulated forms. It was found that for both forms of *B. anthracis*, the decontamination solution showed excellent antimicrobial activity (CFU < 1).

High decontamination factors for BCWA were obtained after only 10 min, accomplishing the requirements imposed by NATO standards. The herein reported new decontamination formulations (neat DS) reached 100% efficacy for *Bacillus anthracis* after 10–15 min, for soman after 20–30 min, and for mustard gas in an interval comprised between 5 and 24 h depending on the type of surface tested. For the neat DS, a decontamination efficiency for HD between 99.2% and 99.98% was obtained after only 10 min. After extreme temperature exposure, we observed a slight increment of the remnant concentration on the surfaces, but the decontamination efficiency was still over 99% on all types of surfaces. GD underwent a faster degradation than HD. For GD, the neat DS exhibited a decontamination efficiency of 98.93% after only 2 min. In contrast with the neat DS, NPs’ suspensions showed faster and more efficient degradation of GD and HD.

The statistical analysis of the decontamination efficiency of HD showed a significant statistical difference between the values of the concentration of each two samples compared to samples involving TiO_2_ formulations; between neat DS and both ZnO nanoparticle formulations; and between zeolite formulations with different concentrations. The comparison between ZnO formulations with different concentrations and between neat DS with zeolite formulations showed no significant difference between the mean values of the HD remnant concentrations.

In the case of GD, the decontamination efficiency of all the sets of statistical comparisons showed no significant difference between the mean values of the concentrations of each of the two compared samples, with the aspect that each of the two nanoparticle formulations (0.5% NPs and 1% NPs) showed a more obvious resemblance between them than when they were compared with neat DS.

The limitations of this study consist of the impossibility of proving the advantages brought on by the nanoparticles in the decontamination of various types of surfaces, such as it being possible for neat DS, due to the particularities of the surfaces tested and also due to the low concentrations of NPs employed for these tests. Therefore, our future work targets include the synthesis of decontamination suspensions with higher concentrations of nanoparticles and also testing the decontamination efficiency against real biological and chemical warfare agents on different types of surfaces to evaluate the relationship between the concentration of NPs and the decontamination performances.

Performing decontamination tests on a real biological warfare agent (*Bacillus anthracis*) and on real chemical warfare agents (soman and sulfur mustard) allowed for an objective evaluation of the performances of the neat decontamination solution, along with the NPs’ influence on the enhancement of the decontamination efficacy. The results of this study are valuable for a proper design of new decontamination formulations.

## Figures and Tables

**Table 1:** Composition of the decontamination solutions.

Sample ID	ZnO (wt.%)	TiO_2_ (wt.%)	Zeolite (wt.%)
DS	0	0	0
DS-ZnO-0.5	0.5	0	0
DS-ZnO-1	1	0	0
DS-TiO_2_-0.5	0	0.5	0
DS-TiO_2_-1	0	1	0
DS-Z-0.5	0	0	0.5
DS-Z-1	0	0	1

**Table 2:** Control ^‡^ of initial contamination level of the tested surfaces.

Microorganism	Painted Metal (CFU/10 cm^2^) *	Glass (CFU/10 cm^2^) *	Rubber (CFU/10 cm^2^) *	Cotton Butyl Rubber (CFU/10 cm^2^) *
*Bacillus anthracis* spores	TMTC **	25 × 10^3^ ± 458.25	15 × 10^3^ ± 55.67	30 × 10^3^ ± 55.67
*Bacillus cereus* spores	TMTC **	25 × 10^3^ ± 86.60	31 × 10^3^ ± 100.00	28 × 10^3^ ± 86.60
*Bacillus subtilis* spores	TMTC **	22 × 10^3^ ± 43.30	27 × 10^3^ ± 91.65	27 × 10^3^ ± 91.65
*Bacillus anthracis*	TMTC **	39 × 10^3^ ± 86.60	26 × 10^3^ ± 43.30	27 × 10^3^ ± 30.00
*Bacillus cereus*	TMTC **	31 × 10^3^ ± 30.00	25 × 10^3^ ± 100.00	25 × 10^3^ ± 50.00
*Bacillus subtilis*	TMTC **	TMTC **	25 × 10^3^ ± 50.00	19 × 10^3^ ± 55.67
*Staphylococcus aureus*	TMTC **	28 × 10^3^ ± 86.60	16 × 10^3^ ± 124.90	27 × 10^3^ ± 91.65
*Pseudomonas aeruginosa*	TMTC **	TMTC **	22 × 10^3^ ± 30.00	18 × 10^3^ ± 124.00

* The initial microbial load was 10^4^ CFU/10 cm^2^, both for spores and vegetative forms. ^‡^ Contamination control was performed by employing pre-moistened sterile swabs for sampling the microorganisms from the contaminated areas (10 cm^2^). The collected samples were allowed to grow on solid culture media. To verify contamination, colony-forming units were counted from the surface of the solid culture media. TMTC ** = too many to count. Sample size (replicates) evaluated for each test was three.

**Table 3:** Decontamination ^‡^ efficiency of the neat organic solution on various types of surfaces for different types of microorganisms.

Microorganism	Painted Metal (CFU/10 cm^2^) *	Glass (CFU/10 cm^2^) *	Rubber (CFU/10 cm^2^) *	Cotton Butyl Rubber (CFU/10 cm^2^) *
*Bacillus anthracis* spores	<1	<1	2 ± 1.00	1 ± 0.00
*Bacillus cereus* spores	3 × 10^2^ ± 2.65	1.0 × 10^2^ ± 8.66	1.1 × 10^2^ ± 8.66	6 × 10^2^ ± 8.66
*Bacillus subtilis* spores	180 ± 5.00	2.0 × 10^2^ ± 8.66	2.2 × 10^2^ ± 7.94	160 ± 2.00
*Bacillus anthracis*	<1	<1	<1	<1
*Bacillus cereus*	TMTC **	2.2 ± 0.72	3.0 ± 1.00	3.5 ± 0.50
*Bacillus subtilis*	TMTC **	10 ± 2.65	1.7 ± 0.26	2.1 ± 0.17
*Staphylococcus aureus*	1.7 ± 0.26	4 ± 0.00	1.1 ± 0.17	2 ± 1.00
*Pseudomonas aeruginosa*	TMTC **	3 ± 1.73	2 ± 1.00	1 ± 0.00

* Values obtained at the completion of the decontamination step (after 10 min of contact with the neat organic decontamination solution). ^‡^ Decontamination control was performed by employing pre-moistened sterile swabs for sampling the microorganisms from the decontaminated areas (10 cm^2^). The collected samples were allowed to grow on solid culture media. To verify decontamination efficiency, colony-forming units were counted from the surface of the solid culture media. TMTC ** = too many to count. Sample size (replicates) evaluated for each test was three.

**Table 4:** Minimal inhibitory concentration (MIC) and minimal bactericidal concentration (MBC) values.

Sample/Microorganism	*E. coli*		*Ps. aeruginosa*		*S. aureus*		*B. spizizenii*	
	MIC (%)	MBC (%)	MIC (%)	MBC (%)	MIC (%)	MBC (%)	MIC (%)	MBC (%)
DS	0.0159	0.0625	0.008	0.25	0.008	0.0159	0.004	0.0159
DS-ZnO-0.5	0.0159	0.0625	0.008	0.25	0.008	0.031	0.004	0.031
DS-ZnO-1	0.008	0.0625	0.008	0.0625	0.004	0.0625	0.004	0.031
DS-TiO_2_-0.5	0.0159	0.0625	0.008	0.25	0.008	0.0625	0.008	0.031
DS-TiO_2_-1	0.0159	0.125	0.008	0.031	0.008	0.0625	0.008	0.031
DS-Z-0.5	0.008	0.25	0.008	0.031	0.008	0.0625	0.004	0.031
DS-Z-1	0.008	0.0625	0.008	0.0159	0.008	0.0159	0.004	0.031

Taking into account that the results were identical between replicates, we did not display the standard deviation that is ±0.

**Table 5:** Decontamination efficiency of neat DS on sulfur mustard (HD) and soman (GD).

Surface Tested	HD Decontamination Efficiency (%)			GD Decontamination Efficiency (%)		
	DS	DS After A1 Cycle (20 … 49 °C)	DS After C1 Cycle (20 … −33 °C)	DS	DS After A1 Cycle (20 … 49 °C)	DS After C1 Cycle (20 … −33 °C)
Painted metal	99.40 ± 0.34	99.20 ± 0.45	99.30 ± 0.41	99.99 ± 0.00	99.99 ± 0.00	99.99 ± 0.00
Rubber	99.20 ± 0.08	99.10 ± 0.78	99.10 ± 0.03	99.98 + 0.08	99.95 ± 0.34	99.98 ± 0.01
Glass	99.98 ± 0.40	100.00 ± 0.00	99.97 + 0.00	100.00 ± 0.00	99.99 + 0.00	100.00 ± 0.00
Cotton butyl rubber	99.40 ± 0.53	99.30 ± 0.52	99.40 ± 0.23	99.93 ± 0.03	99.93 ± 0.07	99.93 ± 0.11
Minimum allowable efficiency *	99.00 ± 0.86	99.00 + 0.76	99.00 ± 0.74	99.90 ± 0.04	99.90	99.90 ± 0.04
Sample size (replicates) evaluated for each surface	3	3	3	3	3	3

* according to NATO standard [37].

## Data Availability

Data is contained within the article.

## Supplementary Materials

The following are available online at https://www.mdpi.com/article/10.3390/ph15010097/s1. Figure S1: EDX mapping results for the nanoparticle clusters: (a,d) ZnO, (b,e) TiO_2_ and (c,f) zeolite; Figure S2: Example of microdilution experiment for establish MIC. *Ps aeruginosa*—MIC determination against nanoparticle solution from sample 4—DS-TiO_2_-0.5 to sample 7—DS-Z-1; Figure S3: Time-kill test verification in liquid media (MHb) for *E. coli* in contact with nanoparticles solution (1–7) and three different contact times (3 h, 6 h and 24 h); Table S1: Statistical data for decontamination degrees (%) of HD obtained for decontamination formulations: neat DS and DS containing 0.5% and 1% ZnO nanoparticles; Table S2: Statistical data for decontamination degrees (%) of HD obtained for decontamination formulations: neat DS and DS containing 0.5% and 1% TiO_2_ nanoparticles; Table S3: Statistical data for decontamination degrees (%) of HD obtained for decontamination formulations: neat DS and DS containing 0.5% and 1% Zeolite nanoparticles; Table S4:Statistical data comparing the HD remnant concentrations after decontamination with neat DS and DS containing ZnO, TiO_2_ and zeolite nanoparticles in two different concentrations; Table S5: Mean value and variance for HD remnant concentration for neat DS and DS containing ZnO, TiO_2_ and zeolite nanoparticles in two different concentrations; Table S6: Statistical data for decontamination degrees (%) of GD obtained for decontamination formulations: neat DS and DS containing 0.5% and 1% ZnO nanoparticles; Table S7: Statistical data for decontamination degrees (%) of GD obtained for decontamination formulations: neat DS and DS containing 0.5% and 1% TiO_2_ nanoparticles; Table S8: Statistical data for decontamination degrees (%) of GD obtained for decontamination formulations: neat DS and DS containing 0.5% and 1% Zeolite nanoparticles; Table S9: Statistical data comparing the GD remnant concentrations after decontamination with neat DS and DS containing ZnO, TiO_2_ and zeolite nanoparticles in two different concentrations; Table S10: Mean value and variance for HD remnant concentration for neat DS and DS containing ZnO, TiO_2_ and zeolite nanoparticles in two different concentrations.
