# Supplementary material for: Antimicrobial Activity and Degradation Ability Study on Nanoparticle-Enriched Formulations Specially Designed for the Neutralization of Real and Simulated Biological and Chemical Warfare Agents
Source: Pharmaceuticals (Basel). 2022 Jan 14;15(1):97. doi: 10.3390/ph15010097 (PMC8778455; doi:10.3390/ph15010097)
Supplement: Supplementary file 1 [file pharmaceuticals-15-00097-s001.zip › pharmaceuticals-1492053-supplementary.pdf]

Supplementary Material

Article

Antimicrobial Activity and Degradation Ability Study on Nanoparticle-Enriched Formulations specially Designed for the Neutralization of Real and Simulated Biological and Chemical Warfare Agents

Raluca-Elena Ginghina[1], Gabriela Toader[2,*], Munizer Purica[3,*], Adriana-Elena Bratu[1], Claudiu Lazaroaie[1], Tudor-Viorel Tiganescu[2], Ramona-Elena Oncioiu[1], George-Ovidiu Iorga[1], Florina-Lucica Zorila[4,5], Mihai Constantin[4], Gabriel Craciun[3], Florin Comanescu[3] and Cosmin Romanitan[3]

[1] Research and Innovation Center for CBRN Defense and Ecology, 225 Oltenitei Ave., Bucharest, 041327, Romania
[2] Military Technical Academy "Ferdinand I", 39-49 George Cosbuc Boulevard, Bucharest, 050141, Romania
[3] National Institute for R&D in Microtechnologies, 126A Erou Iancu Nicolae Street, Voluntari, 077190, Romania
[4] Horia Hulubei National Institute for Physics and Nuclear Engineering, 30 Reactorului Street, 077125 Bucharest, Magurele, Romania
[5] University of Bucharest, Faculty of Biology, Department of Genetics, 91-95 Splaiul Indepententei, 050095 Bucharest, Romania
*Correspondence: Correspondence: nitagabriela.t@gmail.com and munizer.purica@imt.ro

EDX mapping results for the nanoparticle clusters

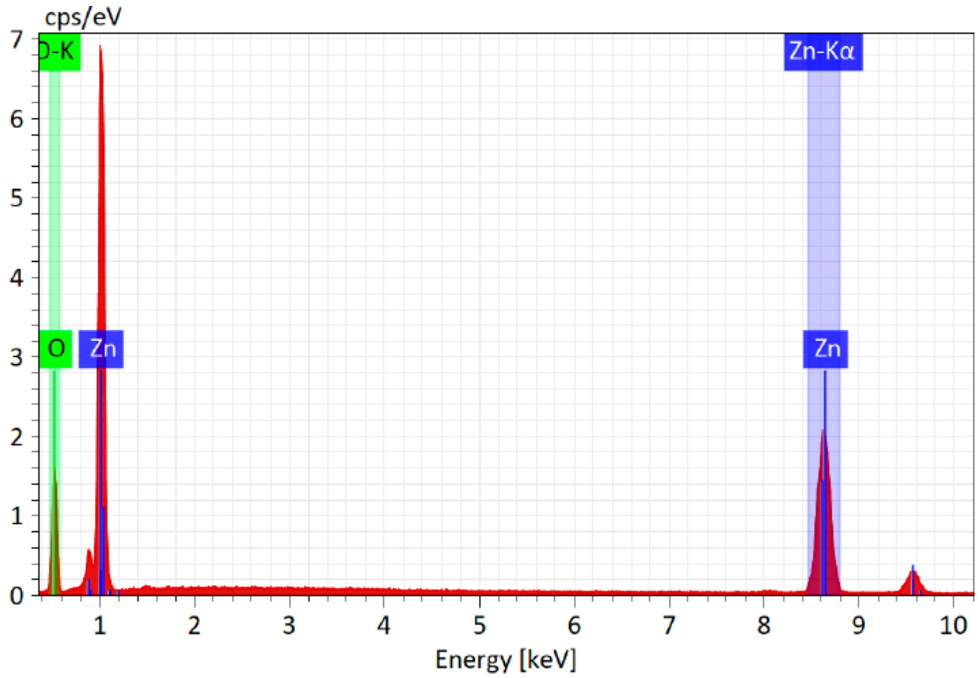

a

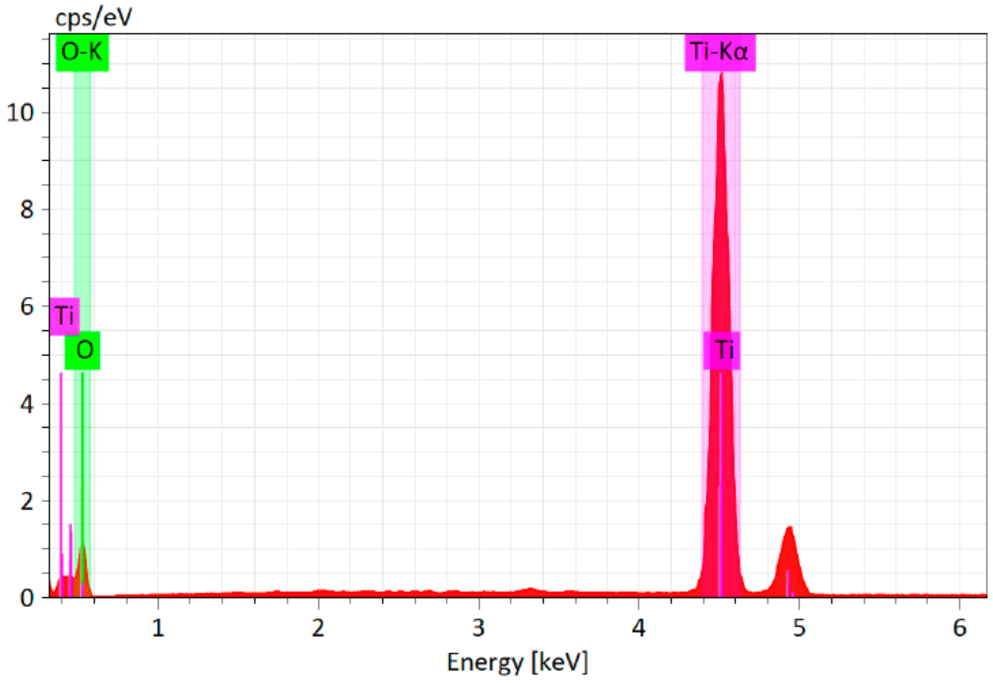

b

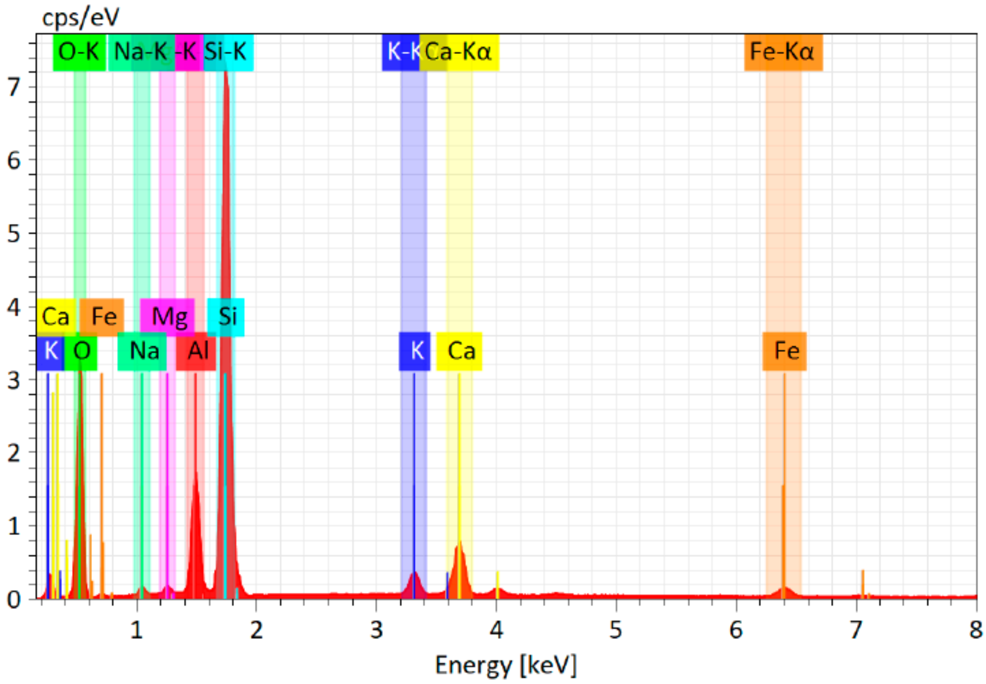

c

Element	At. No.	Netto	Mass [%]	Mass Norm. [%]	Atom [%]	abs. error [%] (1 sigma)	rel. error [%] (1 sigma)
Zinc	30	67560	75,45	63,65	29,99	2,22	2,94
Oxygen	8	13225	43,09	36,35	70,01	2,28	5,28
		**Sum**	**118,55**	**100,00**	**100,00**		

d

Element	At. No.	Netto	Mass [%]	Mass Norm. [%]	Atom [%]	abs. error [%] (1 sigma)	rel. error [%] (1 sigma)
Oxygen	8	23984	75,93	51,27	75,90	4,19	5,51
Titanium	22	576900	72,17	48,73	24,10	1,28	1,78
		**Sum**	**148,10**	**100,00**	**100,00**		

e

Element	At. No.	Netto	Mass [%]	Mass Norm. [%]	Atom [%]	abs. error [%] (1 sigma)	rel. error [%] (1 sigma)
Oxygen	8	124160	81,24	58,60	71,94	4,26	5,24
Silicon	14	415156	37,66	27,16	19,00	1,32	3,51
Aluminium	13	85861	8,94	6,45	4,69	0,38	4,24
Calcium	20	60591	4,99	3,60	1,76	0,10	2,04
Potassium	19	24273	1,82	1,31	0,66	0,04	2,39
Sodium	11	6437	1,66	1,19	1,02	0,12	7,31
Iron	26	13043	1,31	0,94	0,33	0,03	2,15
Magnesium	12	6920	1,03	0,74	0,60	0,06	6,01
		**Sum**	**138,65**	**100,00**	**100,00**		

f

Figure S1. EDX mapping results for the nanoparticle clusters: (a,d) ZnO, (b,e) TiO_2 and (c,f) zeolite

<u>*Antimicrobial activity evaluation*</u>

Minimal inhibitory concentration

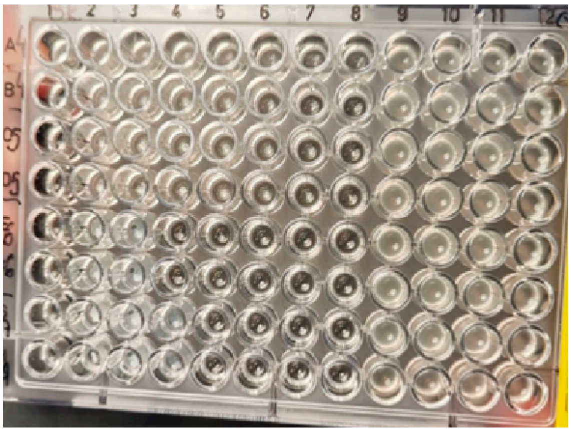

Figure S2. Example of microdilution experiment for establish MIC. Ps aeruginosa - MIC determination against nanoparticle solution from sample 4 - DS-TiO_2-0.5 to sample 7 - DS-Z-1.

All these microplates were visually interpreted because the nanoparticles solutions became turbid and the wells were turbid even in the 5th column, thus being unproper for spectrophotometric reading.

Minimal inhibitory concentration is a quantitative method that determines the minimal concentration needed to inhibit a given microorganism. The readings can be made using a spectrophotometer, by evaluating the absorbance of the sample, only if the tested solution is homogenous and clear. In this case, the obtained absorbance values can be statistically analyzed. If they are not translucent, they can only be evaluated by unaided eye. In our case, due to the enriched nanoparticles suspension used, the spectrophotometric method cannot be applied. Thus, in our case, these readings were made with the unaided eye method, MIC value being the last well without any signs of growth, as described in CLSI (Clinical and Laboratory Standards Institute) and the result is rather qualitative, being basically "+" or "-". The MIC values displayed as a percentage value (%), were obtained by dividing the initial solution, considered 100%, to the dilution factor (2n).

Our setup was organized as follows: The first column was the BK (blank), representing the culture medium (MHb) without microorganisms added and the last column was the Positive control, representing culture medium with appropriate volume of microorganism's suspensions added. The lines were grouped (A and B), (C and D), (E and F) and (G and H), and represent the replicates of the same nanoparticle solution, while the columns represent the binary dilutions starting with ½ concentration in the second column following with the ¼ dilution in the third and so on until 1/1024 in the 11th column. For every nanoparticle solution tested we used two 96-wells plates. We repeated these experiments 4 times.

Time-kill tests

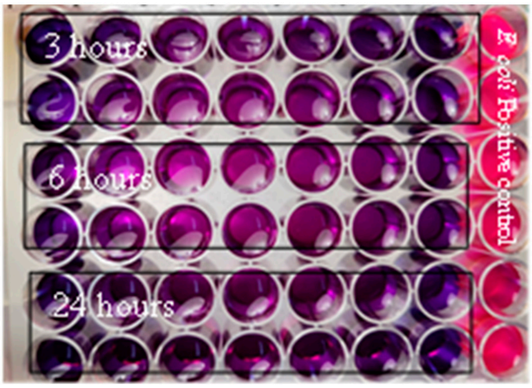

Figure S3. Time -kill test verification in liquid media (MHb) for E. coli in contact with nanoparticles solution (1-7) and three different contact times (3h, 6h and 24 h)

For the time-kill experiment, after first experiment, only few colonies grew. When repeating the experiment, we inoculated in parallel on both MHa and MHb media with the same amount of sample (nanoparticle solution and appropriate microorganism in PBS). For inoculating in liquid media, a 48-well plate was used. Each column represents a different enriched nanoparticle solution, and every 2 lines represents a time of incubation. To observe a possible bacterial metabolism activity, resazurin solution was added to each well (10% of the each well volume). The readings were performed after 2 hours after adding resazurin and incubation. As can be observed, in columns 1 to 7, the tested enriched nanoparticle solutions inhibited metabolic activity of microorganisms in the first 3 h after contact, compared with column 8 that represent the microorganism positive control incubated in the same conditions of the test. Between inoculation periods (after 3 h and 6 h of contact) the 48 well plates were kept at 4-8°C and incubated only after the samples at 24 h of contact were added. This parallel experiment was performed to verify the accuracy of first set of results. For the samples inoculated on MHa the results were similar between repetitions.

Summary of the statistical analysis of the decontamination results

Statistical analysis for mustard gas (HD)

Table S1. Statistical data for decontamination degrees (%) of HD obtained for decontamination formulations: neat DS and DS containing 0.5% and 1% ZnO nanoparticles

	Decontamination degree (%) ± standard deviation						
	2 min	10 min	30 min	1 h	3 h	5 h	24 h
DS	76.42 ± 0.43	77.20 ± 0.41	79.88 ± 0.36	82.84 ± 0.31	92.69 ± 1.32	96.27 ± 0.99	99.99 ± 0
DS-ZnO-0.5	66.13 ± 0.61	67.51 ±0.59	76.85 ± 0.42	84.86 ± 0.27	95.21 ± 1.27	96.71 ± 1.15	100 ± 0
DS-ZnO-1	66.96 ± 0.60	69.64 ± 0.55	78.72 ± 0.38	86.79 ± 0.24	95.73 ± 1.13	97.42 ± 0.90	100 ± 0
Size sample	3	3	3	3	3	3	3

Table S2. Statistical data for decontamination degrees (%) of HD obtained for decontamination formulations: neat DS and DS containing 0.5% and 1% TiO_2 nanoparticles

	Decontamination degree (%) ± standard deviation						
	2 min	10 min	30 min	60 min	3 h	5 h	24 h
DS	76.42 ± 0.43	77.20 ± 0.41	79.88 ± 0.36	82.84 ± 0.31	92.69 ± 1.32	96.30 ± 0.98	99.99 ± 0
DS-TiO2-0.5	94.37 ± 1.01	93.99 ± 1.08	95.45 ± 0.82	96.08 ± 0.71	97.48 ± 0.88	99.03 ± 0.34	100 ± 0
DS-TiO2-1	94.05 ± 1.07	93.47 ± 1.18	94.65 ± 0.96	95.14 ± 0.88	97.27 ± 0.96	98.64 ± 0.48	100 ± 0
Size sample	3	3	3	3	3	3	3

Table S3. Statistical data for decontamination degrees (%) of HD obtained for decontamination formulations: neat DS and DS containing 0.5% and 1% Zeolite nanoparticles

	Decontamination degree (%) ± standard deviation						
	2 min	10 min	30 min	60 min	3 h	5 h	24 h
DS	76.42 ± 0.43	77.20 ± 0.41	79.88 ± 0.36	82.84 ± 0.31	92.69 ± 1.32	96.27 ± 0.99	99.99 ± 0
DS-Z-0.5	94.18 ± 1.05	95.21 ± 1.27	96.47 ± 0.94	96.74 ± 0.86	98.46 ± 0.54	99.29 ± 0.25	100 ± 0
DS-Z-1	94.66 ± 0.96	95.36 ± 1.23	94.68 ± 1.41	96.23 ± 1.00	98.16 ± 0.64	99.19 ± 0.28	100 ± 0
Size sample	3	3	3	3	3	3	3

Table S4. Statistical data comparing the HD remnant concentrations after decontamination with neat DS and DS containing ZnO, TiO_2 and zeolite nanoparticles in two different concentrations.

	DS and DS-ZnO-0.5	DS and DS-ZnO-1	DS-ZnO-0.5 and DS-ZnO-1	DS and DS-TiO2-0.5	DS and DS-TiO2-1	DS-TiO2-0.5 and DS-TiO2-1	DS and DS-Z-0.5	DS and DS-Z-1	DS-Z-0.5 and DS-Z-1

Pearson Correlation	0.988	0.989	0.999	0.975	0.976	0.999	0.974	0.974	0.999
Hypothesized Mean Difference	0	0	0	0	0	0	0	0	0
t Stat	-1.260	-0.738	3.268	3.242	3.195	-3.266	3.323	3.280	-1.068
P(T ≤ t) two-tail	0.248	0.484	0.014	0.014	0.015	0.014	0.013	0.013	0.321
t Critical two-tail	2.365	2.365	2.365	2.365	2.365	2.365	2.365	2.365	2.365
Observations	8	8	8	8	8	8	8	8	8

Table S5. Mean value and variance for HD remnant concentration for neat DS and DS containing ZnO, TiO_2 and zeolite nanoparticles in two different concentrations.

	DS	DS-ZnO-0.5	DS-ZnO-1	DS-TiO2-0.5	DS-TiO2-1	DS-Z-0.5	DS-Z-1
Mean	243.387	265.921	255.919	154.496	158.478	149.571	152.155
Variance	101479.702	104925.427	106081.303	117166.201	116148.435	118473.062	117779.479

Statistical analysis for soman (GD)

Table S6. Statistical data for decontamination degrees (%) of GD obtained for decontamination formulations: neat DS and DS containing 0.5% and 1% ZnO nanoparticles

	Decontamination degree (%) ± standard deviation			
	2 min	10 min	30 min	1 h
DS	98.93 ± 0.40	99.20 ± 0.30	99.60 ± 0.15	99.91 ± 0.04
DS-ZnO-0.5	99.75 ± 0.11	99.95 ± 0.02	100 ± 0	100 ± 0
DS-ZnO-1	99.91 ± 0.04	99.97 ± 0.01	100 ± 0	100 ± 0
Size sample	3	3	3	3

Table S7. Statistical data for decontamination degrees (%) of GD obtained for decontamination formulations: neat DS and DS containing 0.5% and 1% TiO_2 nanoparticles

	Decontamination degree (%) ± standard deviation			
	2 min	10 min	30 min	60 min
DS	98.93 ± 0.40	99.20 ± 0.30	99.60 ± 0.15	99.91 ± 0.04
DS-TiO2-0.5	99.91 ± 0.04	99.96 ± 0.02	100 ± 0	100 ± 0
DS-TiO2-1	99.87 ± 0.06	99.91 ± 0.04	100 ± 0	100 ± 0
Size sample	3	3	3	3

Table S8. Statistical data for decontamination degrees (%) of GD obtained for decontamination formulations: neat DS and DS containing 0.5% and 1% Zeolite nanoparticles

	Decontamination degree (%) ± standard deviation			
	2 min	10 min	30 min	60 min

DS	98.93 ± 0.40	99.20 ± 0.3	99.60 ± 0.15	99.91 ± 0.04
DS-Z-0.5	99.94 ± 0.03	99.98 ± 0.01	100 ± 0	100 ± 0
DS-Z-1	99.93 ± 0.03	99.95 ± 0.02	100 ± 0	100 ± 0
Size sample	3	3	3	3

Table S9. Statistical data comparing the GD remnant concentrations after decontamination with neat DS and DS containing ZnO, TiO_2 and zeolite nanoparticles in two different concentrations.

	DS and DS-ZnO-0.5	DS and DS-ZnO-1	DS-ZnO-0.5 and DS-ZnO-1	DS and DS-TiO2-0.5	DS and DS-TiO2-1	DS-TiO2-0.5 and DS-TiO2-1	DS and DS-Z-0.5	DS and DS-Z-1	DS-Z-0.5 and DS-Z-1
Pearson Correlation	0.999	0.999	0.999	0.999	0.999	0.999	0.999	0.999	0.999
Hypothesized Mean Difference	0	0	0	0	0	0	0	0	0
t Stat	2.475	2.365	1.152	2.365	2.393	-1.616	2.344	2.350	-1.372
P(T<=t) two-tail	0.069	0.077	0.313	0.077	0.075	0.181	0.079	0.079	0.242
t Critical two-tail	2.776	2.776	2.776	2.776	2.776	2.776	2.776	2.776	2.776
Observations	5	5	5	5	5	5	5	5	5

Table S10. Mean value and variance for HD remnant concentration for neat DS and DS containing ZnO, TiO_2 and zeolite nanoparticles in two different concentrations.

	DS	DS-ZnO-0.5	DS-ZnO-1	DS-TiO2-0.5	DS-TiO2-1	DS-Z-0.5	DS-Z-1
Mean	204.720	200.600	200.240	200.260	200.440	200.160	200.240
Variance	197660.977	199701.175	199880.153	199870.158	199780.383	199920.068	199880.113
